# Acute Sarcopenia: Systematic Review and Meta‐Analysis on Its Incidence and Muscle Parameter Shifts During Hospitalisation

**DOI:** 10.1002/jcsm.13662

**Published:** 2024-12-17

**Authors:** Luke Aldrich, Theocharis Ispoglou, Konstantinos Prokopidis, Jasem Alqallaf, Oliver Wilson, Antonis Stavropoulos‐Kalinoglou

**Affiliations:** ^1^ Carnegie School of Sport Leeds Beckett University, Headingley Campus Leeds UK; ^2^ Institute of Life Course and Medical Sciences University of Liverpool Liverpool UK

**Keywords:** atrophy, cachexia, disuse, wasting, weakness

## Abstract

**Background:**

Acute sarcopenia is sarcopenia lasting less than 6 months, typically following acute illness or injury. It may impact patient recovery and quality of life, advancing to chronic sarcopenia. However, its development and assessment remain poorly understood, particularly during hospitalisation. This systematic review aimed to elucidate the incidence of acute sarcopenia and examine changes in muscle parameters during hospitalisation.

**Methods:**

Eighty‐eight papers were included in the narrative synthesis; 33 provided data for meta‐analyses on the effects of hospitalisation on handgrip strength (HGS), rectus femoris cross‐sectional area (RFCSA) and various muscle function tests. Meta‐regressions were performed for length of hospital stay (LoS) and age for all meta‐analyses; sex was also considered for HGS.

**Results:**

Acute sarcopenia development was assessed in four studies with a pooled incidence of 18% during hospitalisation. Incidence was highest among trauma patients in intensive care (59%), whereas it was lower among medical and surgical patients (15%–20%). Time of development ranged from 4 to 44 days. HGS remained stable during hospitalisation (SMD = 0.05, 95% CI = −0.18:0.28, *p* = 0.67) as did knee extensor strength. LoS affected HGS performance (θ = 0.04, 95% CI = 0.001:0.09, *p* = 0.045) but age (*p* = 0.903) and sex (*p* = 0.434) did not. RFCSA, reduced by 16.5% over 3–21 days (SMD = −0.67, 95% CI = −0.92:−0.43, *p* < 0.001); LoS or time between scans did significantly predict the reduction (θ = −0.04, 95% CI = −0.077:−0.011, *p* = 0.012). Indices of muscle quality also reduced. Muscle function improved when assessed by the short physical performance battery (SMD = 0.86, 95% CI = 0.03:1.69, *p* = 0.046); there was no change in 6‐min walk (*p* = 0.22), timed up‐and‐go (*p* = 0.46) or gait speed tests (*p* = 0.98). The only significant predictor of timed up‐and‐go performance was age (θ = −0.11, 95% CI = −0.018:−0.005, *p* = 0.009).

**Conclusions:**

Assessment and understanding of acute sarcopenia in clinical settings are limited. Incidence varies between clinical conditions, and muscle parameters are affected differently. HGS and muscle function tests may not be sensitive enough to identify acute changes during hospitalisation. Currently, muscle health deterioration may be underdiagnosed impacting recovery, quality of life and overall health following hospitalisation. Further evaluation is necessary to determine the suitability of existing diagnostic criteria of acute sarcopenia. Muscle mass and quality indices might need to become the primary determinants for muscle health assessment in hospitalised populations.

## 1 Background + Introduction

Acute sarcopenia refers to the rapid deterioration of muscle health, developing secondary to a medical event, such as an illness or injury [1]. In intensive care units (ICUs), muscle wasting may be associated with prolonged length of stay and increased mortality [2, 3, 4]. After discharge, acute sarcopenia may predispose people to developing chronic sarcopenia, the long‐term progressive age‐related decline of muscle health. Chronic sarcopenia is known to compromise quality of life, independence and overall health as well as increase the risk of other comorbidities, frailty and mortality [5, 6, 7, 8]. Therefore, accurate identification of acute sarcopenia is of paramount importance.

An attempt to outline the diagnosis and characteristics of acute sarcopenia was made by the European Working Group on Sarcopenia in Older People (EWGSOP2) [1]. Acute and chronic sarcopenia share common diagnostic criteria within muscle health, such as strength, mass, quality and function, but acute sarcopenia lasts 6 months or less [1]. However, it is unclear if all aspects of muscle health deteriorate at the same rate and if some are more sensitive to acute changes.

Further, the diagnosis of sarcopenia is based on muscle parameters reaching specific thresholds rather than a rate of change or magnitude of reduction. For example, a male patient's handgrip strength (HGS) may decrease from 50 kg at hospital admission to 30 kg at discharge, a 40% reduction. However, this does not meet the EWGSOP2 threshold of < 27 kg, which is necessary to classify as probable sarcopenia risk [1]. These thresholds, which are based on normative data from studies involving predominantly healthy older adults [1, 9], may not accurately reflect the risk of severity of sarcopenia in diverse, clinically distinct populations.

This reliance on static thresholds fails to capture the nuances of acute muscle health deterioration in people experiencing medical emergencies, which can impede effective management and treatment [10]. Acute sarcopenia may develop very rapidly; Welch et al. [11] suggested that muscle health may deteriorate within 28 days of an illness or injury. Indeed, in the first week of ICU stay, people may experience a daily loss of 2% in muscle mass amounting to > 10% losses within a week [12].

The precise mechanisms underpinning acute sarcopenia are unclear; however, the extent of muscle mass reduction in ICU patients is more pronounced than in healthy individuals exposed to bed rest and limb suspension [13]. This suggests that there is an interplay between muscle protein synthesis and degradation [14], inflammation [15], hormonal changes and mitochondrial dysfunction [2] in acute sarcopenia development and muscle atrophy in a clinical setting. Inevitably, this requires a refined approach to sarcopenia diagnosis and management in this setting.

Acute sarcopenia is often associated with chronic diseases and symptoms such as low‐grade inflammation and hormonal imbalances, which induce a catabolic state within the muscle [2]. Other causes could include immobilisation from injury, leading to muscle degradation through reduced mechanical loading [13, 14], mitochondrial dysfunction and insulin resistance [16, 17]. These molecular changes ultimately lead to the accelerated onset of more obvious morphological and physical changes experienced during hospitalisation, that is, reduced muscle mass, strength, quality and function.

Understanding the onset of acute sarcopenia in different hospitalised patients is an urgent priority. By achieving this, we can develop accurate definitions and criteria, which in turn will inform the design and implementation of effective strategies to mitigate the condition for individuals subjected to hospitalisation. With these considerations in mind, the objectives of this systematic review and meta‐analysis are to

- Determine the incidence of acute sarcopenia development in hospitalised patients,
- Understand the changes that occur to different muscle parameters that are involved in sarcopenia assessments in specific hospitalised patients.

## 2 Methods

This systematic review was conducted according to the Preferred Reporting Items for Systematic Reviews and Meta‐Analyses (PRISMA) criteria [18]. The review received PROSPERO accreditation with the complete protocol for this research available for viewing (CRD42022329996).

### 2.1 Data Sources and Searches

An exhaustive search strategy (supplementary material) was devised to identify studies that investigated or observed parameters of acute sarcopenia resulting from illness or injury in populations with or recovering from disease. Studies only included hospitalised populations.

Search terms were applied to both title and abstract fields using the following databases: PubMed, Cochrane Library, Embase, CINAHL, APA PsycInfo and SPORTDiscus. Filters for ‘Humans’, ‘English’ and ‘Adults’ were applied. No filters were applied to publication date. The results were exported to EndNote 20 (Clarivate) to aid in organising and filing. Only primary studies were included in the synthesis. Pertinent reviews in the field, identified through the search strategy, had their reference lists reviewed for potential eligibility of studies not captured by the search strategy.

### 2.2 Inclusion and Exclusion Criteria

Inclusion criteria were as follows: patients with or recovering from disease in hospital, patients with acute illness or injury requiring hospitalisation, assessments of muscle strength, mass, quality or function at a minimum of two different time points during hospitalisation, intervention studies with a relevant control group and observational cohort studies.

Exclusion criteria were as follows: adolescents or children (aged < 18 years), athletic populations and injuries, only one time point assessment of muscle parameters, cohorts with pre‐existing sarcopenia at the first/baseline assessment or where acute and chronic sarcopenia could not be differentiated, first/baseline assessment obtained > 4 days since hospital admission and cohorts with cancer or similar condition requiring chemotherapy treatment.

For studies assessing sarcopenia, we included all with any recognised or validated definition or assessment methods [i.e., EWGSOP1, EWGSOP2, Foundation for the National Institutes of Health, Asian Working Group for Sarcopenia (AWGS) 1, AWGS2, International Working Group for Sarcopenia (IWGS), Baumgartner, Mourtzakis, Morley, Sarcopenia Definitions and Outcomes Consortium (SDOC) and Delmonico]. Rapid sarcopenia development was determined based on the diagnosis of sarcopenia within current definitions, but within the 6‐month period outlined by the EWGSOP2 [1]. Studies that measured HGS, knee extensor strength (KES), muscle mass, quality or function were also included.

### 2.3 Data Extraction and Quality Assessment

**FIGURE 1:**
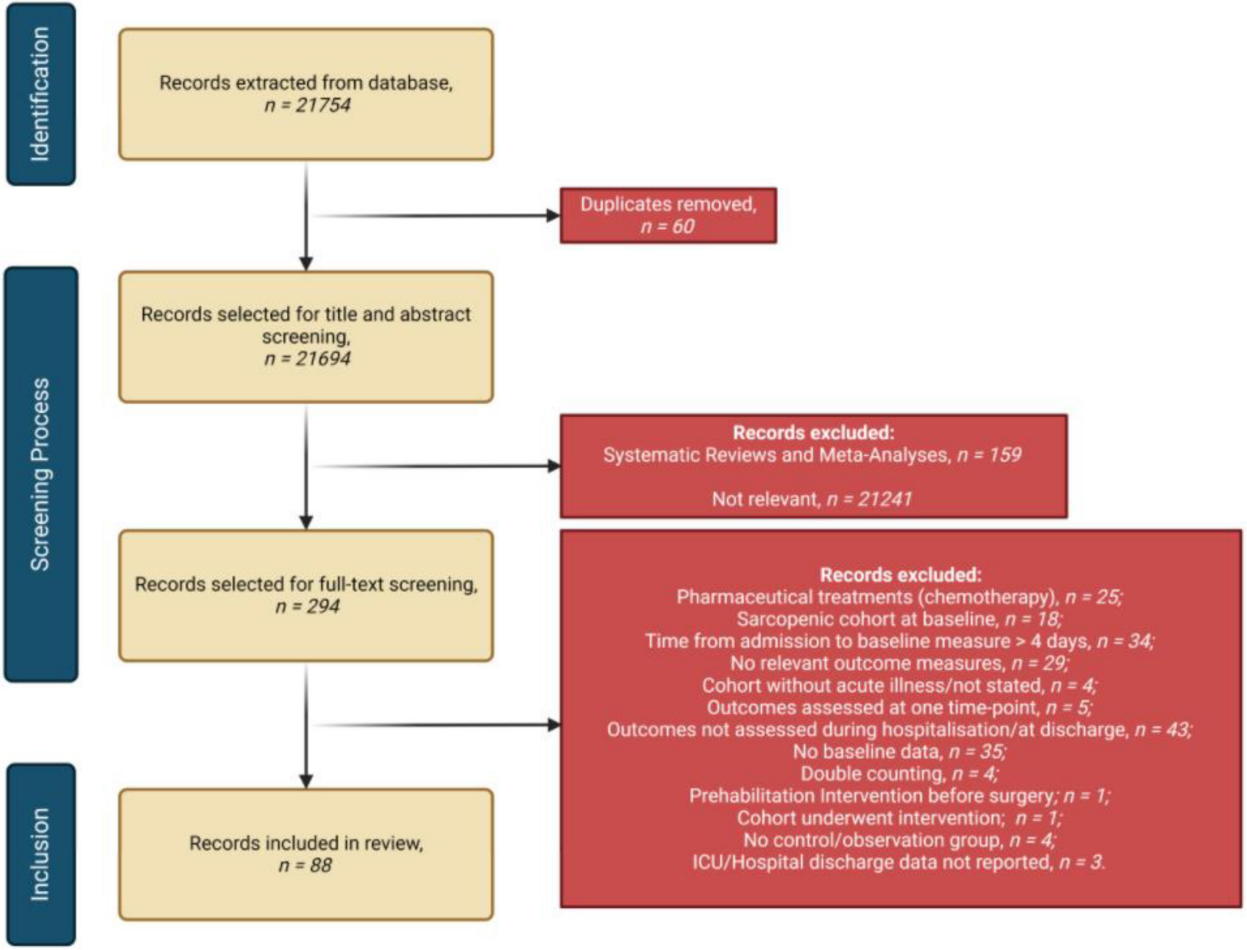
Flow diagram outlining the identification and screening process according to PRISMA [18] guidelines.

Two investigators (LA and KP) independently evaluated titles and abstracts of all initial results to identify studies for full‐text review. In case of uncertainty, the article underwent a full‐text review for a comprehensive understanding. The same investigators independently conducted full‐text reviews. Any disagreements were resolved through discussion and if consensus could not be reached, a third reviewer (ASK) was consulted. The screening process is outlined in Figure 1.

Data extraction was conducted using the Cochrane tool for data extraction (The Cochrane Collaboration, v.3) with additional relevant criteria added. Key criteria included study information, methodological details and sarcopenia muscle parameters. Extraction for all studies was performed in duplicate (LA and KP).

Studies were assessed for bias (LA and KP) using the National Heart, Lung and Blood Institute tools appropriate for each study design (National Heart, Lung and Blood Institute, Study Quality Assessment Tools, NHLBI, 2021. *Accessed: 16.01.2023*).

### 2.4 Data Synthesis and Analysis

A narrative synthesis was performed with tabular synthesis where appropriate. To interpret results, data were categorised based on muscular parameters assessed, assessment tools used, hospitalised patient groups (e.g., COVID‐19 and trauma) and their exposures (e.g., ICU and medical ward).

Mean and standard deviations (SD) and 95% confidence intervals (CI) are reported where appropriate. Where sufficient data were available, that is, when a minimum of three studies per variable provided clear data for admission and discharge/follow‐up during hospitalisation, meta‐analyses were conducted for quantitative analysis.

Statistical analyses were performed using package metafor in R version 4.2 (R Foundation for Statistical Computing, Vienna, Austria). A random‐effects model was used to determine overall effect size using preobservation to postobservation values to calculate standardised mean difference (SMD) and 95% confidence intervals (CIs) [19].

CIs and test statistics were calculated via a *t*‐distribution using the Hartung–Knapp–Sidik–Jonkman approach [20]. Effect sizes were interpreted as trivial (< 0.2), small (0.20–0.49), moderate (0.50–0.79) and large (> 0.79), according to Cohen's recommendations [21].

Meta‐analysis results are presented in forest plots containing SMD and CIs, significance (*p*‐value set at < 0.05) and heterogeneity assessed by the *I* ^2^ statistic. Heterogeneity was considered unimportant (0%–40%), moderate (30%–60%), substantial (50%–90%) and considerable (75%–100%), according to Cochrane recommendations [22].

Meta‐regression analyses were performed to identify predictors of change in SMD, such as the duration of hospital stay and sex. If a meta‐analysis included more than one outcome measure from the same study, effect estimates were nested within studies using a multilevel structure [23]. Where meta‐analyses were performed with only three studies and there was large heterogeneity, sensitivity analyses were performed by removal of each study to ascertain whether this would affect the model parameters.

## 3 Results

### 3.1 Study Inclusion and Risk of Bias

The final searches, conducted on 18.06.2024, yielded 21 754 potentially eligible articles. After removal of duplicates, 21 694 remained for screening of titles and abstracts. Relevant systematic reviews and meta‐analyses were also searched to identify further relevant studies. Utilising rigorous inclusion and exclusion criteria, 294 publications remained for full‐text review. Of those, 206 were excluded (Figure 1), leaving 88 articles for synthesis.

Eighty‐eight studies included for synthesis involved 9476 patients, from 38 randomised controlled trials (RCTs) and 50 observational cohort studies, whose data were extracted as part of the selection criteria. The specific age range of included patients is unclear; however, the mean and median ages of patients within included studies spanned from 33 to 88 years. All included studies took place in a hospitalised setting with information provided in Table 1. The overall risk of bias for the RCTs and observational cohort studies were low and high, respectively (Tables S1 and S2).

**TABLE 1:** Included studies and their characteristics.

Author/reference	Year of publication	Study design	Sample size	Nature of exposure	Length of hospital stay (days)	Acute sarcopenia assessed?	HGS assessed?	Knee extensor strength assessed?	Muscle mass assessed?	Muscle function assessed?	Muscle quality assessed?
Aarden et al. [24]	2021	Prospective, observational cohort study	*N* = 343	Medical wards (CV, GI, pulmonary, infection, other)	5.7 (IQR: 3.9–8.7)	✗	✓	✗	✓	✓	✗
Ahmad et al. [25]	2024	Randomised controlled trial	*N* = 15	Surgical wards (heart valve surgery)	10.64 ± 3.79	✗	✗	✗	✗	✓	✗
Annetta et al. [26]	2017	Prospective, observational cohort study	*N* = 20	ICU Wards (Trauma)	ICU = 22 (IQR: 16–37)	✗	✗	✗	✓	✗	✗
Attaway et al. [27]	2022	Retrospective analysis of prospective cohort study	COVID‐19 Cohort: *N* = 95, Alive: *N* = 79, Died: *N* = 16	ICU and medical wards (COVID‐19)	Alive = 13.0 (IQR, 6.0, 28.0). Died = 21.0 (IQR, 13.8, 37.3)	✗	✗	✗	✓	✗	✗
Ballesteros‐Pomar et al. [28]	2021	Prospective, observational cohort study	*N* = 200	Medical wards (cardiorespiratory, digestive, nephrological, oncological)	7 (IQR: 7)	✓	✓	✓	✓	✓	✗
Bao et al. [29]	2022	Randomised controlled trial	*N* = 20	ICU Wards (pelvic fracture, thoracolumbar fracture, multiple rib fractures, haemorrhagic shock, lung infection, other)	12.2 ± 4.18	✗	✗	✗	✓	✗	✗
Barbalho et al. [30]	2019	Within‐patient randomised trial	*N* = 20	ICU wards	11 ± 2.2	✗	✗	✗	✓	✗	✗
Beyer et al. [31]	2011	Randomised controlled trial	*N* = 14	Medical wards (acute infection)	24.5 (19–64)	✗	✓	✗	✓	✗	✗
Bodilsen et al. [32]	2013	Prospective, observational cohort study	*N* = 33	Medical wards (CV, GI, pulmonary, infection, other)	7.5 (IQR: 4.25–11.00)	✗	✓	✓	✗	✓	✗
Bologna & Pone [33]	2022	Retrospective analysis of randomised and observational trial	*N* = 40	ICU wards (COVID‐19)	20.1	✗	✓	✗	✓	✗	✗
Borges et al. [34]	2016	Randomised controlled trial	*N* = 19	CABG surgery	8.3 ± 2.2	✗	✗	✗	✗	✓	✗
Borges et al. [15]	2020	Prospective, observational cohort study	*N* = 45	ICU wards (severe sepsis or septic shock)	20 (11.0–31.5 (25th–75th percentile))	✗	✓	✗	✓	✗	✗
Bradford et al. [35]	2023	Retrospective, observational cohort study	*N* = 81	ICU Wards (Trauma)	38 ± 21	✗	✗	✗	✓	✗	✗
Butera et al. [36]	2023	Retrospective, observational cohort study	*N* = 697	Medical and surgical wards (joint arthroplasty, pneumonia, heart failure, COPD, hip fracture)	18 ± 12.1	✗	✗	✗	✗	✓	✗
Chapple et al. [37]	2022	Randomised controlled trial	*N* = 42	ICU wards (CV, respiratory, GI, neurological, sepsis, trauma, other)	< 60 days	✗	✗	✗	✓	✗	✗
Chites et al. [38]	2021	Prospective, observational cohort study	*N* = 600	Medical, surgical and ICU wards	NR	✗	✓	✗	✗	✗	✗
da Silva et al. [39]	2022	Prospective, longitudinal cohort study	*N* = 41	ICU wards (burns)	24 (IQR: 16–40)	✗	✓	✗	✗	✗	✗
De Andrade‐Junior et al. [40]	2021	Prospective, observational cohort study	*N* = 32	ICU wards (COVID‐19)	21 (15–42 (range))	✗	✓	✗	✓	✗	✓
de Buyser et al. [41]	2014	Prospective, observational cohort study	*N* = 639	Medical wards (hypertension, ischaemic heart disease, heart failure, type II diabetes, osteoarthritis, COPD, renal failure)	9 (IQR: 6–14)	✗	✓	✗	✗	✓	✗
de Carvalho et al. [42]	2022	Prospective, observational cohort study	*N* = 1168	Medical and surgical wards	Men = 7.86 ± 10.28; Female = 7.33 ± 9.61	✗	✓	✗	✗	✗	✗
de Morton et al. [43]	2007	Randomised controlled trial	*N* = 126	Medical wards (respiratory, circulatory, digestive, genitourinary, other)	6 (IQR: 3.25–9.75) days	✗	✗	✗	✗	✓	✗
de Moura et al. [44]	2023	Prospective, observational cohort study	*N* = 30	ICU wards (COVID‐19)	7	✗	✗	✗	✓	✗	✗
Dimopoulos et al. [3]	2020	Prospective, observational cohort study	*N* = 165	ICU wards (Cardiac surgery)	5	✗	✗	✗	✓	✗	✗
Dirks et al. [45]	2015	Observational, clinical trial	*N* = 9	ICU wards (comatose)	Not reported	✗	✗	✗	✓	✗	✗
Dusseaux et al. [46]	2019	Prospective, observational cohort study	*N* = 25	ICU wards (Medical: septic shock, sepsis, acute severe pancreatitis, cardiac arrest, pneumonia, endocarditis)	10.9 ± 4.7	✗	✗	✗	✓	✗	✓
Eibel et al. [47]	2022	Randomised controlled trial	*N* = 6	Surgical wards (CABG)	7	✗	✗	✗	✗	✓	✗
Fränzel et al. [48]	2024	Randomised controlled trial	*N* = 29	Medical wards (various)	21	✗	✗	✗	✗	✓	✗
Gerovasili et al. [49]	2009	Prospective, observational controlled trial	*N* = 13	ICU wards (sepsis, trauma, neurologic, other)	Not reported	✗	✗	✗	✓	✗	✗
Giangregorio et al. [50]	2009	Randomised controlled trial	*N* = 7	Surgical wards (hip fracture)	26.1 ± 17.6	✗	✗	✗	✗	✓	✗
Gil et al. [51]	2022	Prospective, observational, cohort study	*N* = 80	Medical and ICU wards (COVID‐19)	8 (IQR: 5–12)	✗	✓	✗	✓	✗	✗
Gualtieri et al. [52]	2020	Prospective, observational, cohort study	*N* = 30	ICU wards (COVID‐19)	Not reported	✗	✗	✗	✓	✗	✓
Hadda et al. [53]	2018	Prospective, observational, cohort study	*N* = 70	ICU wards (sepsis)	7	✗	✗	✗	✓	✗	✗
Haines et al. [54]	2019	Prospective, observational cohort study	*N* = 107	ICU wards (trauma)	39.00 [19.00, 65.50] (Median (IQR)); ICU = 11.33 [5.54, 25.14] (Median (IQR))	✓	✗	✗	✓	✗	✗
Hasanloei et al. [55]	2021a	Randomised controlled trial	*N* = 50	ICU wards (CV, trauma, GI, respiratory, CNS)	44.28 ± 9.31	✗	✗	✗	✓	✗	✗
Hasanloei et al. [56]	2021b	Randomised controlled trial	*N* = 20	ICU wards (trauma)	19.15 ± 8.46 (ICU = 13.35 ± 5.55)	✗	✗	✗	✓	✗	✗
Hayes et al. [57]	2018	Prospective, observational cohort study	*N* = 25	ICU wards (medical & surgical: various)	20	✗	✗	✗	✓	✗	✓
Hickson et al. [58]	2004	Randomised controlled trial	*N* = 300	Medical wards (various)	23.0 (IQR: 14–39)	✗	✓	✗	✗	✗	✗
Hu et al. [59]	2020	Randomised controlled trial	*N* = 50	Medical wards (infection, cardiac, respiratory, GI, neurological, endocrine, other)	6.92 ± 4.08	✗	✓	✗	✗	✓	✗
Jones et al. [60]	2006	Randomised controlled trial	*N* = 80	Medical wards (various)	11 (IQR: 6–21) days	✗	✗	✗	✗	✓	✗
Kangalgil et al. [61]	2022	Prospective, observational cohort study	*N* = 35	ICU wards (surgical & trauma)	7	✗	✗	✗	✓	✗	✗
Kangalgil et al. [62]	2024	Prospective, observational cohort study	*N* = 44	ICU wards (various	7	✗	✗	✗	✓	✗	✗
Karlsen et al. [63]	2017	Prospective, observational cohort study	*N* = 151	Medical wards (respiratory, infection, MSK, genitourinary, digestive, endocrine, neurological, other)	3–13	✗	✓	✗	✗	✓	✗
Katari et al. [64]	2018	Prospective, observational, cohort study	*N* = 100	Surgical and medical ICU patients	NR	✗	✗	✗	✓	✗	✗
Kim et al. [65]	2023	Prospective, observational, cohort study	*N* = 58 (hip fracture); *N* = 57 (hip disease)	Surgical wards (hip fracture). Medical wards (hip disease)	Hip fracture = 10.2. Hip disease = 8.7	✗	✓	✗	✗	✗	✗
Kouw et al. [66]	2019	Prospective, observational cohort study	*N* = 26	Surgical wards (THA)	5.6 ± 0.3	✗	✓	✗	✓	✗	✓
Kronborg et al. [67]	2017	Randomised, assessor‐blinded effectiveness study	*N* = 45	Surgical wards (hip fracture)	11.8 ± 6.8	✗	✗	✓	✗	✓	✗
Lee et al. [4]	2021	Prospective, observational cohort study	*N* = 86	Medical and surgical ICU wards (CV, respiratory, GI, neurological, sepsis, trauma, metabolic, renal, immunocompromised)	17.43 (IQR: 10.69–36.68)	✗	✗	✗	✓	✗	✓
López Jiménez et al. [68]	2024	Prospective, observational cohort study	*N* = 98	Medical wards (heart failure, coronary artery disease, pulmonary infection, urinary infection)	7.6 ± 4.3	✗	✓	✗	✓	✗	✗
Martín‐Salvador et al. [69]	2015	Prospective, observational cohort study	> 75 years old *N* = 48; < 75 years old *N* = 68	Medical wards (pneumonia)	> 75 years old 8.05 ± 3.1. < 75 years old 8.33 ± 3.9	✗	✓	✓	✗	✓	✗
Martínez‐Velilla et al. [70]	2019	RCT	*N* = 185	Medical wards (CV, infection, pulmonary, GI, neurological, other)	8 (IQR: 4)	✗	✓	✗	✗	✓	✗
Martone et al. [71]	2017	Observational cohort study	*N* = 394	Medical wards (heart disease, diabetes, stroke, other)	10.2 ± 8.1	✓	✓	✗	✓	✓	✗
Mayer et al. [72]	2020	Prospective, observational, cohort study	*N* = 41	ICU wards (medical or cardiothoracic)	11.2 (IQR: 8–19), ICU = 8 (4)	✗	✓	✓	✓	✓	✓
McNelly et al. [73]	2020	Multicentre, interventional, single‐blind RCT	*N* = 121	ICU wards (multiorgan failure)	22.8 (IQR: 1.5–183)	✗	✗	✗	✓	✗	✗
Mets et al. [74]	2004	Single‐blind, prospective, randomised controlled trial	*N* = 15	Medical wards (acute infection with high circulating inflammation)	Not reported	✗	✓	✗	✗	✗	✗
Mgbemena et al. [75]	2022	Prospective, observational cohort study	*N* = 101	Surgical wards (cardiac)	11 ± 5	✗	✓	✗	✗	✗	✗
Nakanishi et al. [76]	2020	Multicentre, prospective, observational, cohort study	*N* = 64	Surgical and medical ICU wards (respiratory, sepsis, cardiac)	38 (IQR: 18–57)	✗	✗	✗	✓	✗	✗
Nery et al. [77]	2012	Controlled trial	*N* = 15	Surgical wards (lung resection)	Not reported	✗	✗	✗	✗	✓	✗
Neto et al. [78]	2024	Randomised controlled trial	*N* = 30	ICU wards (COVID‐19)	4.66 ± 2.99	✗	✓	✗	✗	✗	✗
Nickels et al. [79]	2020	Single‐blinded, RCT	*N* = 36	ICU wards (sepsis, trauma, cardiac, GI, pneumonia, vascular, airway obstruction, overdose, other)	17.9 (IQR: 13.0, 29.4)	✗	✓	✗	✓	✓	✗
Norheim et al. [80]	2017	Prospective, observational, cohort study	Continuous inflammation: *N* = 138. Became noninflammatory: *N* = 76	Medical wards (acute illness with high circulating inflammation)	CI = 11.0 (IQR: 8–15). BNI = 8.0 (IQR: 6–11)	✗	✓	✗	✗	✓	✗
Ogasawara et al. [81]	2018	Randomised controlled trial	*N* = 21	Medical wards (COPD)	12.1 ± 3.9	✗	✗	✗	✓	✗	✗
Ortiz‐Alonso et al. [82]	2019	Randomised controlled trial	*N* = 125	Medical wards (respiratory, circulatory, renal/neurologic, CNS, digestive)	7 (IQR: 5)	✗	✗	✗	✗	✓	✗
Parry et al. [83]	2015	Prospective, observational, cohort study	*N* = 22	ICU wards (medical and surgical)	22.0 (IQR: 12.8–43.5)	✗	✗	✗	✓	✗	✓
Pitta et al. [84]	2006	Prospective, observational, cohort study	*N* = 17	Medical wards (COPD)	8	✗	✗	✓	✓	✗	✗
Pourhassan et al. [85]	2020	Prospective, longitudinal, observational, cohort study	*N* = 41	Medical wards (falls and fractures, pneumonia, OA, stroke, UTI)	16 (14–18)	✗	✓	✓	✓	✗	✗
Puthucheary et al. [86]	2013	Prospective, observational, cohort study	*N* = 63	ICU wards (sepsis, trauma, acute liver failure, cardiogenic shock)	30 (11–334)	✗	✗	✗	✓	✗	✗
Ramsey et al. [87]	2022	Longitudinal, observational, cohort study	*N* = 572	Medical wards (MSK, neurological, infection, CV, respiratory, other)	19.8 (IQR: 13.6–28.9)	✗	✓	✗	✗	✓	✗
Raymond et al. [88]	2017	Randomised controlled trial	*N* = 232	Medical wards (fractures, falls, respiratory, renal, cardiac, neurological, dementia, MSK, other)	12.2 (95% CI: 11–13.5)	✗	✗	✗	✗	✓	✗
Rodrigues et al. [89]	2021	Prospective, observational, cohort study	*N* = 60	ICU wards (respiratory, sepsis, cardiac, coma, other)	34.5 (25%–75% quartile, 17–54)	✗	✗	✗	✓	✗	✗
Segaran et al. [90]	2017	Prospective, observational, pilot study	*N* = 44	ICU wards (medical, surgical, trauma)	Not reported	✗	✓	✗	✓	✗	✗
Segers et al. [91]	2021	Randomised controlled trial	*N* = 47	ICU wards (abdominal, cardiac, GI, respiratory, organ transplant, thoracic, other)	Not reported	✗	✗	✓	✓	✗	✗
Silva et al. [92]	2019	Randomised controlled trial	*N* = 30	ICU wards (TBI)	42 (95% CI, 20–56)	✗	✗	✗	✓	✗	✓
Strasser et al. [93]	2023	Randomised controlled trial	*N* = 10	Medical wards (arthrosis, MSK)	7	✗	✓	✗	✓	✗	✓
Tazerout et al. [94]	2022	Retrospective cohort study	*N* = 114	ICU wards (trauma)	ALL = 29 (19–45 (median IQR). Low APTMA = 19 (IQR: 15–29). Moderate APTMA = 29 (IQR: 20–39). High APTMA = 48 (IQR: 31–61)	✗	✗	✗	✓	✗	✗
Temporiti et al. [95]	2022	Randomised controlled trial	*N* = 40	Surgical wards (THA)	4	✗	✗	✗	✗	✓	✗
Toledo et al. [96]	2021	Prospective, observational cohort study	*N* = 74	ICU wards (sepsis, stroke, lung transplant, cardiac insufficiency)	7	✗	✗	✗	✓	✗	✗
Torres‐Sanchez et al. [97]	2017	Randomised controlled trial	*N* = 29	Medical wards (COPD)	10.38 ± 2.47	✗	✗	✓	✗	✓	✗
Trung et al. [98]	2019	Prospective, observational, cohort study	*N* = 80	ICU wards (tetanus infection)	25 (IQR: 20–33)	✗	✗	✗	✓	✗	✗
Turton et al. [99]	2016	Prospective, observational, cohort study	*N* = 22	ICU wards (various)	10	✗	✗	✗	✓	✗	✓
Verceles et al. [100]	2023	Randomised controlled trial	*N* = 23	ICU wards (respiratory, cardiac, neurological, other)	14	✗	✗	✗	✓	✗	✗
Vermeeren et al. [101]	2004	Randomised controlled trial	*N* = 24	Medical wards (COPD)	8	✗	✓	✓	✓	✗	✗
Welch et al. [102]	2022a	Prospective, observational cohort study	*N* = 79	Surgical and medical wards (emergency surgery, colorectal surgery, infection)	Not reported	✓	✓	✗	✓	✓	✓
Welch et al. [103]	2022b	Prospective, observational cohort study	*N* = 79	Surgical and medical wards (emergency surgery, colorectal surgery, infection)	Not reported	✓	✓	✗	✓	✓	✓
Werner et al. [104]	2024	Prospective, observational cohort study	*N* = 107	Medical wards (MSK, neurological, infection, CV, GI, other)	20.2 ± 5.8	✗	✓	✗	✗	✓	✗
Wnuk et al. [105]	2016	Single‐blind RCT	*N* = 16	Surgical wards (abdominal aortic aneurysm)	6.56 ± 0.73	✗	✗	✗	✗	✓	✗
Wu et al. [106]	2023	Multicentre RCT	*N* = 28	ICU wards (lung disease, sepsis, CKD, acute pancreatitis, other)	13.05 (IQR: 7.25–28.25)	✗	✓	✗	✗	✓	✗
Xie et al. [107]	2019	Prospective, observational, cohort study	*N* = 95, ICU‐AW Group: *N* = 50. Non‐ICU‐AW Group: *N* = 45	ICU wards (pneumonia, COPD, pulmonary, ARDS)	ICU‐AW = 20.54 ± 6.37; NON‐ICU‐AW = 17.76 ± 4.93	✗	✗	✗	✓	✗	✗
Xue et al. [95, 108]	2022	Randomised controlled trial	*N* = 43	Surgical wards (cardiac surgery)	8.16 ± 1.13	✗	✗	✗	✗	✓	✗

Abbreviations: APTMA = acute post‐trauma muscle atrophy; ARDS = acute respiratory distress syndrome; BNI = became noninflammatory; CABG = coronary artery bypass graft; CI = continuous inflammation; CKD = chronic kidney disease; CNS = central nervous system; COPD = chronic obstructive pulmonary disease; CV = cardiovascular; FL = fascicle length; GI = gastrointestinal; HGS = handgrip strength; ICU = intensive care unit; ICU‐AW = intensive care unit–acquired weakness; IQR = interquartile range; KES = knee extensor strength; LoS = length of hospital stay; MSK = musculoskeletal; NR = not reported; OA = osteoarthritis; PA = pennation angle; RCT = randomised controlled trial; TBI = traumatic brain injury; TBI = traumatic brain injury; THA = total hip arthroplasty; UTI = urinary tract infection; VI = vastus intermedius; VL = vastus lateralis.

### 3.2 Acute Sarcopenia Development

Four studies specifically assessed the development of sarcopenia during a period of hospitalisation [28, 54, 71, 102].

In a large cohort study, Martone et al. [71] observed sarcopenia development in 14.7% (58 out of 394) patients over a mean period of 5.4 ± 6.7 days. Sarcopenia was determined according to EWGSOP1 criteria [109]. Over half of these patients also experienced a reduction of more than 10% of muscle mass. Using updated EWGSOP2 criteria [1], another large‐scale study identified 19% of a medical cohort developed sarcopenia over 7 days of hospitalisation [28]. Furthermore, Welch et al. [102, 103] reported 20% of their cohort developed sarcopenia, whereas an additional 8% developed probable sarcopenia within an average period of 7 ± 2 days. Only 10% of their cohort showed no reductions in any muscle parameters. Haines et al. [54] identified variable rates of sarcopenia development (8%–59%), over 4–44 days in ICU trauma patients, using abdominal CSA as a determinant of muscle mass and sarcopenia diagnosis.

When pooling the data from these studies together, 126 out of 708 hospitalised patients (18%) developed sarcopenia during a hospital stay ranging from 4 to 44 days.

### 3.3 Muscle Strength

#### 3.3.1 Handgrip Strength

Twenty‐nine studies assessed changes in HGS during hospitalisation (Table S3). The response of HGS to hospitalisation was variable: Some reported a reduction in HGS [33, 39, 40, 42, 51, 69, 70, 78], whereas others observed an increase [39, 41, 58, 65, 69, 80, 87, 93, 106]. These changes typically occurred within a period of 5–30 days. Six studies found no change in HGS [31, 66, 74, 80, 101, 104].

**FIGURE 2:**
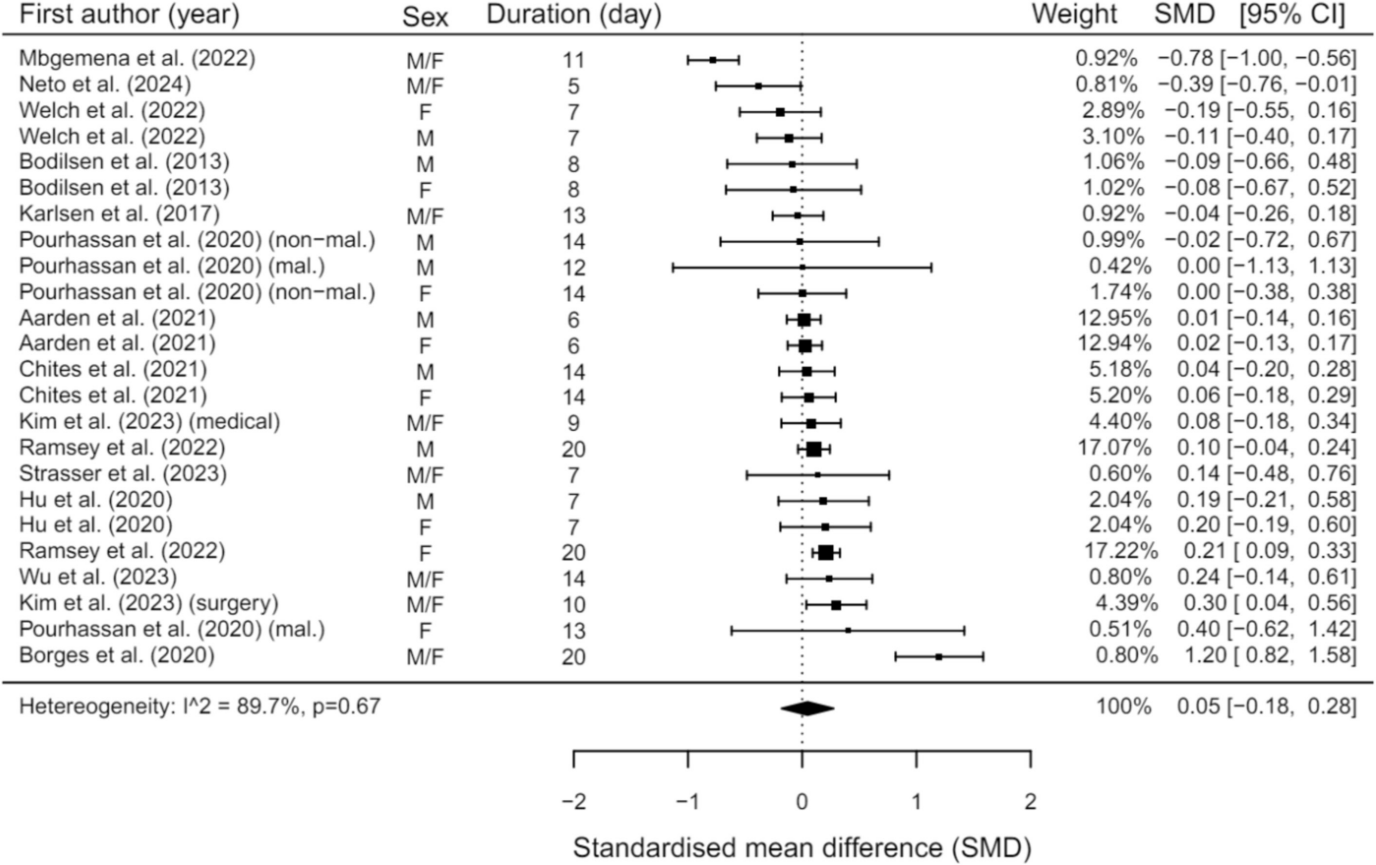
Meta‐analysis illustrating changes in HGS during hospitalisation (F = Female; M = Male; mal. = malnourished; non‐mal. = non‐malnourished).

Furthermore, we conducted a meta‐analysis on the studies that provided sufficient data (*n* = 14). There was no significant change in HGS with a trivial effect size (SMD = 0.05, 95% CI = −0.18:0.28, *p* = 0.67; Figure 2). Meta‐regression analyses revealed that length of stay was a significant predictor of change for HGS (θ = 0.04, 95% CI = 0.001:0.09, *p* = 0.045). The age and sex of patients were not predictors of change for HGS (*p* = 0.903 and *p* = 0.434, respectively). It is crucial to note the considerable degree of heterogeneity between these studies (*I* ^2^ = 89.69%, *p* < 0.0001).

#### 3.3.2 Knee Extensor Strength

Eight studies assessed KES (Table S4). Four of these identified a reduction in KES during hospitalisation lasting 8–14 days [69, 84, 85, 97]. In contrast, other studies observed no change [91] or increased [32, 67, 101] KES within 7–10 days.

### 3.4 Muscle Mass

#### 3.4.1 Whole–Body Muscle Mass

Eight studies presented findings for whole‐body muscle mass, typically assessed by skeletal muscle mass (SMM) either as a percentage of body mass or via the skeletal muscle index (SMI) although, some studies expressed this as lean body mass index (LBMI) or fat‐free mass (FFM) (Table S5). The majority of studies observed a decline in whole‐body muscle mass [55, 56, 81, 101, 102] between 7 and 36 days across various exposures and illnesses in medical wards, ICU and a combination of both. Some studies observed no change [24, 84] and nonsignificant increases [81, 93] during 6–21 days of hospitalisation. Of these studies, one identified a nonsignificant increase in muscle mass when reported as LBMI, but when measured by SMI, this showed a nonsignificant decrease [81]. The methods of measurement across studies assessing whole‐body muscle mass were BIA and isotopic potassium.

#### 3.4.2 Muscle Mass of Specific Muscles

Table S6 details findings from 12 studies that investigated muscle mass changes in the erector spinae, pectoralis, L3 and L4 psoas, abdominal muscles, biceps brachii, adductor pollicis, forearm and thigh muscles. All studies demonstrated declines in muscle mass in medical, surgical and ICU patients [27, 35, 37, 44, 46, 52, 53, 54, 76, 89, 90, 94]. Within one of these studies, the highest incidence of sarcopenia development was observed at 59% in ICU trauma patients over 44 days [54]. Two subgroups within this same study were the only exceptions where no change was observed in abdominal muscle mass in line with the L3 psoas region although there were significant reductions in the L4 psoas muscle [38]. These two subgroups had either died or were in ICU on day 10 after hospital admission or had been discharged from ICU and were alive on day 10 [38].

#### 3.4.3 Rectus Femoris Cross‐Sectional Area (RFCSA)

**FIGURE 3:**
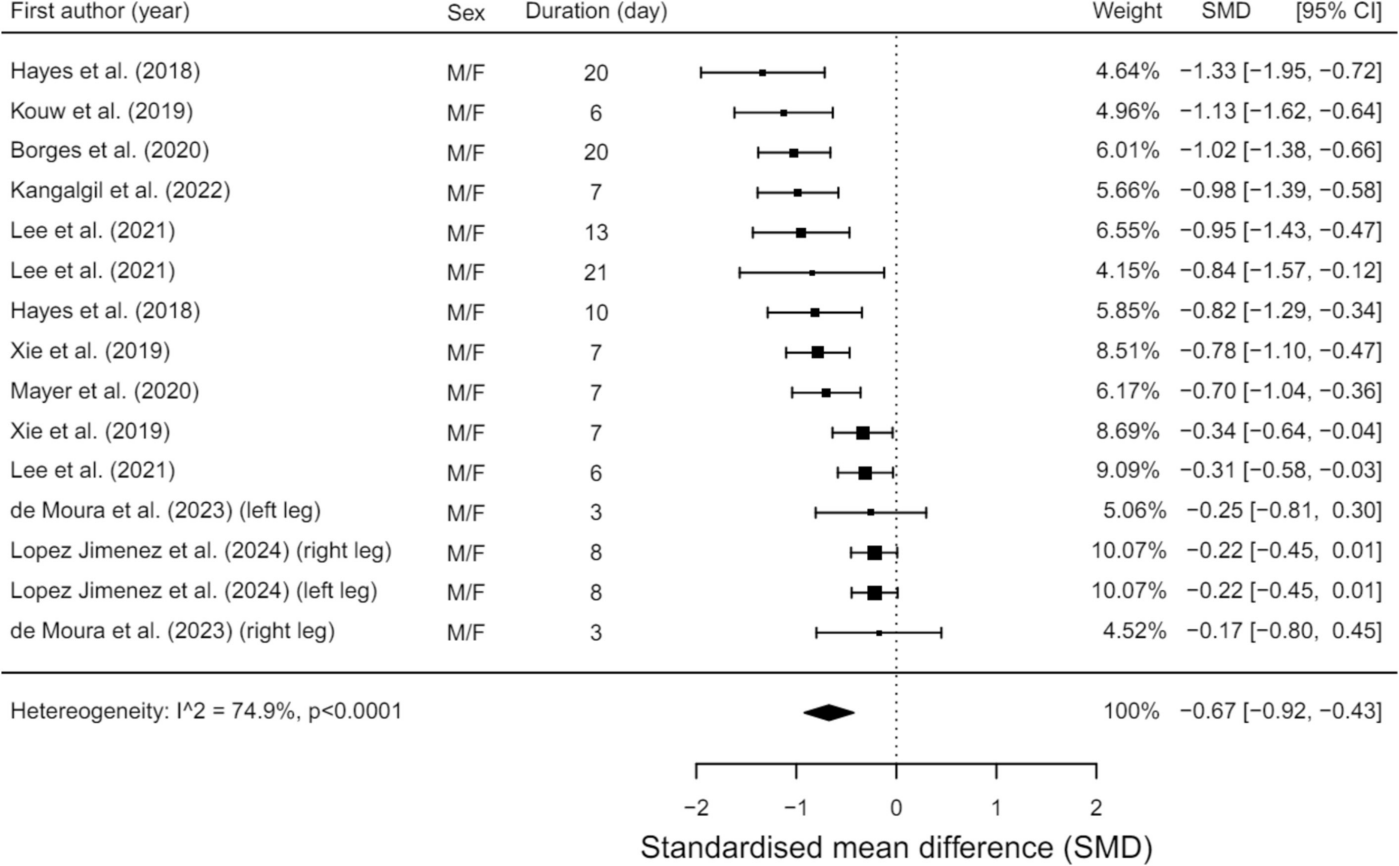
Meta‐analysis illustrating changes in RFCSA during hospitalisation.

A meta‐analysis was conducted on studies that offered sufficient data regarding changes in RFCSA during hospitalisation (*n* = 9). Analysis revealed significant reductions in RFCSA with a moderate effect size (SMD = −0.67, 95% CI = −0.92:−0.43, *p* < 0.001; Figure 3). This equates to a 16.5% reduction in RFCSA between 3 and 21 days. Ultrasound and CT scans were the tools utilised for this assessment. Furthermore, a meta‐regression showed hospital length of stay or duration between scans in that exposure did statistically influence the changes in RFCSA (θ = −0.04, 95% CI = −0.077:−0.011, *p* = 0.012), whereas age did not (*p* = 0.129). It is imperative to note the substantial heterogeneity within these studies (*I* ^2^ = 74.94%, *p* < 0.001).

#### 3.4.4 Muscle Mass of Lower Limbs

Thirty‐five studies assessed changes in lower limb muscle mass (Table S7). Assessment measures included thickness, volume and CSA of specific muscle groups such as the quadriceps, mid‐thigh region, vastus lateralis, vastus intermedius, tibialis anterior and RF. Techniques employed were ultrasound, CT and MRI scans, with some studies using muscle biopsies to measure muscle fibre CSA.

All studies reported some degree of reduction in muscle mass of the lower limbs [3, 4, 15, 26, 29, 30, 33, 37, 40, 44, 45, 49, 51, 53, 57, 61, 62, 64, 66, 68, 72, 73, 76, 79, 83, 85, 86, 89, 91, 92, 96, 98, 99, 100, 107] over periods ranging from 6 to 60 days across diverse hospital exposures and illnesses. Kouw et al. [66] observed a decrease in muscle mass assessed by CT scans, but muscle biopsy data highlighted an increase in Type I and II muscle fibre CSA over 5.6 days.

### 3.5 Muscle Function

Muscle function changes during hospitalisation were assessed in 26 studies (Table S8). Various tools measured muscle function, including the SPPB, TUG, 6MWT, chair stand test (CST) and gait speed. Meta‐analyses were able to be conducted for the SPPB, TUG, 6MWT and gait speed during hospitalisation. Muscle function declined in seven studies across the 6MWT, TUG and gait speed, predominantly related to a surgical context [25, 34, 47, 69, 77, 95, 105].

In studies not related to surgical procedures, muscle function remained stable or improved [24, 32, 59, 63, 70, 72, 80] over 3–13 days.

Meta‐analyses were conducted where data were sufficient for SPPB (*n* = 4), 6MWT (*n* = 5), TUG (*n* = 6) and gait speed (*n* = 3). Analyses revealed significant improvements in the SPPB (SMD = 0.86, 95% CI = 0.03:1.69, *p* = 0.046) with neither length of stay nor age being significant predictors (*p* = 0.51 and *p* = 0.36, respectively). There were no significant changes to 6MWT, TUG or gait speed during hospitalisation (*p* = 0.22, *p* = 0.46 and *p* = 0.98, respectively). However, meta‐regressions revealed age as a significant predictor for TUG (θ = −0.11, 95% CI = −0.018:−0.005, *p* = 0.009). It is important to note the substantial heterogeneity between the studies for the SPPB (*I* ^2^ = 95.51%, *p* < 0.001), 6MWT (*I* ^2^ = 94.3%, *p* < 0.001), TUG (*I* ^2^ = 96.23%, *p* < 0.001) and gait speed (*I* ^2^ = 98.8%, *p* < 0.001).

**FIGURE 4:**
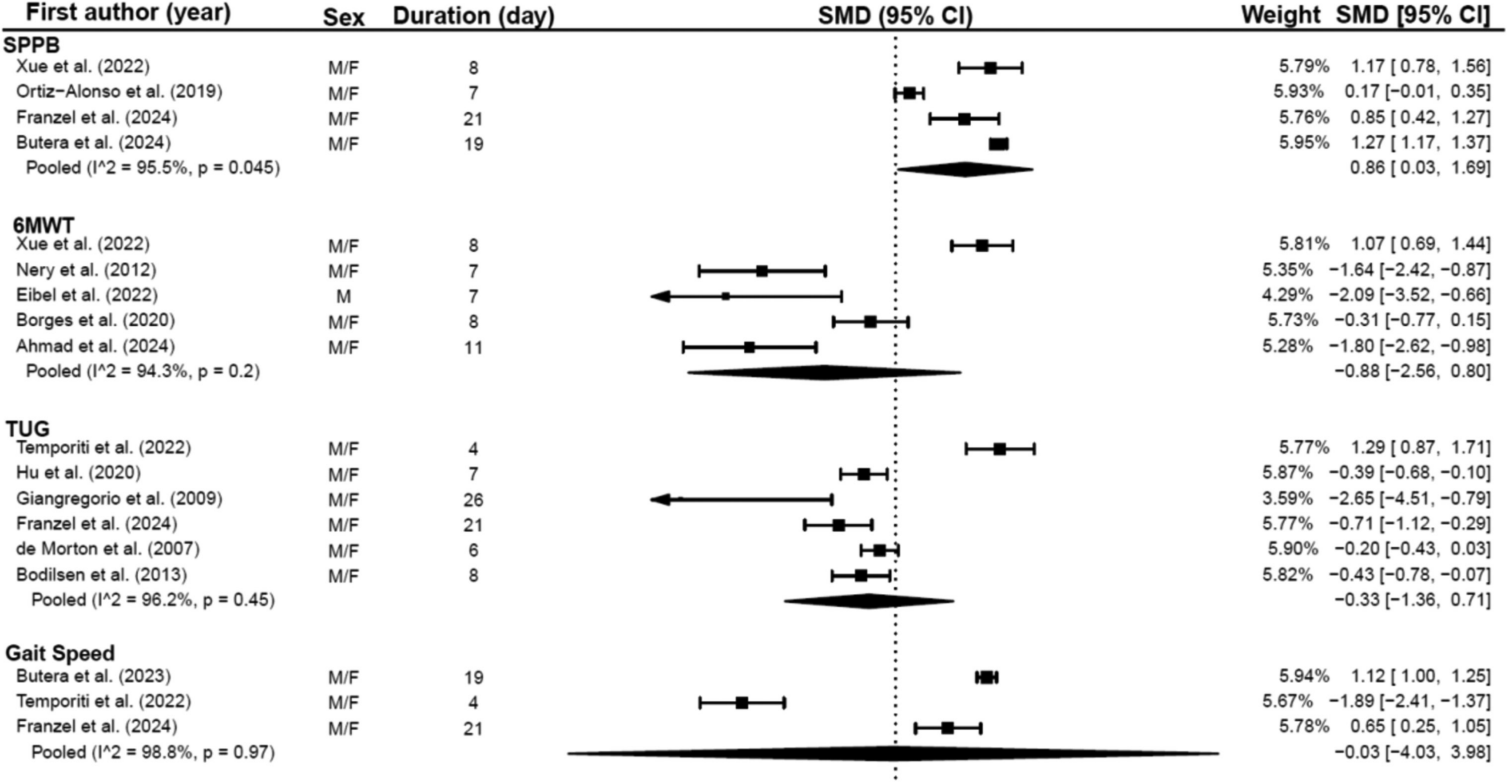
Meta‐analysis illustrating changes in muscle function during hospitalisation.

Sensitivity analyses were performed for the gait speed meta‐analysis. Analyses showed that removing Butera et al. [36] (SMD = −0.62, 95% CI = −16.733:15.501, *p* = 0.712, *I* ^2^ = 98.27%, *p* < 0.001), Fränzel et al. [48] (SMD = −0.37, 95% CI = −19.521:18.778, *p* = 0.846, *I* ^2^ = 99.19%, *p* < 0.001) and Temporiti et al. [95] (SMD = 0.93, 95% CI = −2.065:3.916, *p* = 0.159, *I* ^2^ = 80.02%, *p* = 0.025) individually did not significantly influence overall changes in gait speed (Figure 4).

### 3.6 Muscle Quality Indices

Twelve studies provided data for various muscle quality indices, utilising methods such as ultrasound, CT scans and BIA (Table S9). The indices measured included echogenicity, pennation angle, fascicle length, muscle attenuation and phase angle. Echogenicity showed an increase—indicating a reduction in muscle quality—in nearly all patient groups [40, 57, 72, 83, 92]. A decline in muscle attenuation was also observed in specific patient groups via CT scan [46, 52, 66]. Reductions were also observed in phase angle [93, 102, 103]. No changes were observed in muscle attenuation in obese patients [52] or in echogenicity in only one study [4]. Lee et al. [4] also identified an improvement in fascicle length; however, this coincided with a reduction in pennation angle. Another study identified reductions in pennation angle and fascicle length [99]. These alterations were observed over periods spanning 6–22 days.

## 4 Discussion

This systematic review and meta‐analysis aimed to determine the incidence of acute sarcopenia development and identify the changes that occur to muscle strength, mass, quality and function within hospitalised patients. The key findings of this review are (1) specific assessment of acute sarcopenia is very rarely conducted; (2) when assessed, its overall incidence is 18% but it varies considerably between groups of patients and possibly due to differences in methods of assessment; (3) it can develop rapidly, in as quickly as 4 days; and (4) muscle mass and quality indices, especially in the lower limbs, are more affected than upper limb strength or overall muscle function during hospitalisation. Our findings suggest that HGS and muscle function assessments may not be suitable for evaluating acute changes in muscle health in hospital settings. Relying on these tests may lead to underdiagnosis of acute muscle health deterioration and impact patient well‐being during and after hospitalisation.

Assessment of sarcopenia, according to EWGSOP2 criteria [1], entails tests for several muscle parameters including strength, mass, quality and function. Commonly, HGS or CST are used to identify probable sarcopenia, which is confirmed by assessments of muscle mass or quality. Finally, severity is determined by muscle function tests. This process is rarely followed in clinical settings, and in most cases, assessment of muscle health is done by evaluating only one of the above‐named parameters. Indeed, only four [28, 54, 71, 102] of the included studies assessed sarcopenia specifically. It is not clear why this is, but the complexity and time demands of conducting all tests coupled with the clinical condition of the patients may be relevant. Furthermore, the novelty of acute sarcopenia and the recent pandemic halting many research practices, such as gaining ethical approval and research grants [110], may mean that there have been delays in performing relevant studies.

The incidence of sarcopenia differed between studies. This is likely due to the presenting illness at hospital admission—trauma patients in ICU had a higher incidence than medical or surgical patients. Different methodological approaches may have also contributed. For example, the highest incidence was reported by Haines et al. [54], who used abdomen muscle CSA to determine sarcopenia [111]. HGS is used as the primary determinant of sarcopenia in the most recent EWGSOP [1] criteria. Interestingly, this is one of the main changes compared with the former version where the primary determinant was muscle mass [109]. Studies comparing the two EWGSOP criteria for identifying sarcopenia in the same population consistently conclude that the EWGSOP1 [109] criteria return a higher sarcopenia prevalence than the EWGSOP2 [1] criteria [112, 113]. It therefore seems that muscle mass is more sensitive than muscle strength, and although this may result in several false positive cases for chronic sarcopenia, it may enhance the identification of people suffering from acute changes in muscle health and requiring suitable management strategies to attenuate this.

Our meta‐analysis further supports that, as we showed that HGS was not affected by length of hospitalisation. This is surprising especially when coupled with significant reductions in muscle mass. However, given the various presenting conditions and physical capacity of patients upon hospital admission, initial HGS assessments are unlikely to represent maximal effort. Previous studies highlighted differences in submaximal and maximal HGS scores in community‐dwelling and hospitalised patients and sarcopenia prevalence [114, 115]. One study indicated a near halving of sarcopenia prevalence based on submaximal HGS scores [114], potentially due to weakness on arrival and improved strength following treatment in hospital. Our findings support these studies [114, 115] and challenge the use of HGS as a standalone diagnostic criterion for sarcopenia in hospitalised settings.

Like HGS, muscle function assessments may not be suitable to diagnose acute sarcopenia in nonsurgical populations, as it requires effort from the patient. Patient mobility and functional capacity are likely to be limited upon hospital admission for reasons related to their condition rather than muscle health. Successful hospital treatment is likely to improve function to a greater extent than any detriment because of potential muscle losses. Specific muscle function tests have been used at hospital discharge, and lower functionality is associated with worse outcomes postdischarge [116]. Given the mental and physical fatigue and reduced motivation a patient may experience while recovering from illness in hospital, muscle assessments that require a level of effort to produce their ‘best’ performance may be influenced by these feelings. Therefore, it remains unclear whether such tests can truly reflect muscle health and function in hospitalised patients.

Conversely, our findings suggest that muscle mass and indices of muscle quality are reduced during hospitalisation, particularly in the lower limbs. Muscle mass and quality assessments differ to HGS and muscle function as they are objective and cannot be influenced by patient effort. Similarly to muscle mass, muscle quality indices were negatively affected, evidenced by unfavourable changes in muscle echogenicity and pennation angle in the quadriceps, muscle attenuation in the abdomen and erector spinae, and phase angle at a whole‐body level. Although it is important to consider the variability in the measurement tools and the findings, this does provide some support for previous research that has promoted the prognostic importance of muscle quality indices in hospitalised patients, identifying that critical illness and surgical exposures are significantly associated with lower muscle quality [117, 118]. Muscle quality indices are becoming increasingly reported during hospitalisation as the assessment methods become more readily available, for example, using ultrasound. However, there is no criterion for any muscle quality indices in the EWGSOP2 definition for sarcopenia unlike other muscle parameters.

Changes in muscle parameters occur rapidly, regardless of age, and timely identification of muscle health deterioration is important. However, this is not the case in all hospitalised populations, and underlying health conditions would undoubtedly have an impact on the rate of change to muscle parameters. For example, populations in ICUs may experience higher inflammatory profiles promoting a more catabolic state. Fazzini et al. [12] identified rapid reductions in quadriceps muscle mass of approximately 2% per day in ICU. Hardy et al. [13] identified rapid muscle atrophy during the early stages of immobilisation with variable rates of atrophy in different muscles. Quadriceps and triceps surae showed the greatest rates of atrophy, particularly in critically ill patients versus healthy volunteers subjected to bed rest [13]. Such changes in muscle health have been reported to affect hospital length of stay, risk of complications and in‐hospital mortality [2, 3, 4, 76]; however, the long‐term effects relating to the clinical significance, reversibility and changes within specific morbidities remain unknown with further research required.

Interestingly, our meta‐regression results for HGS, RFCSA and all muscle function tests except TUG showed that age was not a predictor of change in these muscle parameters. This is surprising given that older populations are generally considered more vulnerable to the impact of acute illness on muscle health than younger adults. Our review included adults younger than 30 years old, demonstrating that individuals across all age groups are susceptible to reductions in muscle health during hospitalisation. Therefore, the incidence of acute sarcopenia and the observed reductions in muscle mass and quality may accelerate ageing across the entire adult population, potentially inducing conditions such as frailty, which has previously been identified in older adults [103].

Despite the above evidence, a timeline for the development of acute sarcopenia or for its optimal monitoring has not been established. Welch et al. [11] defined acute sarcopenia as changes in muscle mass and function developing within 28 days from a medical event. This timeline seems appropriate based on our findings and those mentioned previously [12, 13]. However, EWGSOP2 [1] suggests that acute sarcopenia is resolved within 6 months. The rationale for this is unclear as the impact of such rapid deteriorations of muscle health is unknown and may have dire consequences on patient well‐being. Furthermore, there are no specific directions on when the baseline is to be established or when follow‐up assessments should be conducted.

Finally, all definitions of sarcopenia base its diagnosis on muscle parameters reaching a specific threshold. Welch et al. [102] identified that, in addition to the number of participants developing acute sarcopenia, an extra 23% of participants lost over 10% of at least one other muscle parameter. Such reductions, even though not sufficient to classify sarcopenia, are likely to predispose them to significant health risks and further muscle health concerns in the long term [119]. This poses a question around the use of specific thresholds for identifying acute sarcopenia and whether the rate or magnitude of change should be considered.

### 4.1 Limitations

Our study is limited by methodological variability in the included studies, evidenced by high heterogeneity in the meta‐analyses. The heterogeneity was influenced by different measurement tools, variations in age, underlying health conditions and other demographic factors. These variables may influence our findings and could explain the inconsistencies we identified, thus questioning the comparability of our results. Furthermore, very few studies specifically assessed sarcopenia, even though all assessed at least one of the relevant muscle parameters. Often, the assessment of these parameters was a secondary outcome measure, and there was frequent underreporting of predata and postdata, as well as the omission of specific numbers of the cohort who experienced a decrease or increase in the muscle parameter in question. This limited our ability to perform additional meta‐analyses and to attain a more thorough understanding of changes to muscle parameters during hospitalisation. Moreover, there is no set time for optimal muscle assessments during hospitalisation, so the time between assessments was variable, limiting our ability to establish a clear timeline for changes in muscle parameters.

The included studies also contained patients experiencing different hospital exposures. For example, patients were admitted to multiple exposures during one stay where measures of muscle health were obtained, such as ICU and medical wards. This diversity in patients made grouping these patients challenging and was not feasible in our analyses, meaning our results are difficult to extrapolate.

Additionally, although the search strategy devised was extensive and identified key papers in the area, a limitation of our study is the lack of further search terms, which may have resulted in missed related keywords (e.g., ‘hospitalis*’ may be more appropriate than using ‘hospitalization’). This could potentially affect the comprehensiveness of the search strategy.

## 5 Conclusions and Future Directions

Sarcopenia can develop rapidly during the early stages of hospitalisation. Of the key parameters of muscle health involved in determining sarcopenia, we identified that muscle mass and quality are reduced during hospitalisation and muscle strength and function are not. This finding suggests that there may be widespread underdiagnosis of sarcopenia during hospitalisation and many patients may be discharged with compromised muscle health and greater risk of developing chronic sarcopenia. Therefore, sarcopenia assessments in clinical settings could be more reliable if objective assessments are prioritised early in hospitalisation, that is, muscle mass and muscle quality indices. Also, consideration needs to be given to the magnitude of reductions.

Our findings serve as a steppingstone towards fostering better collaboration and translational research for enhanced clinical practice in diagnosing and treating acute sarcopenia. However, methodological inconsistencies and the paucity of data suitable for meta‐analyses limit our study. These limitations highlight current challenges while simultaneously laying the groundwork for future investigations. Future research should consider the limitations we have highlighted and focus on understanding the long‐term effects of rapid muscle health deterioration on quality of life and postdischarge functional independence. Muscle quality indices should be considered seriously for incorporation into diagnostic criteria. Addressing these areas will deepen understanding of acute sarcopenia and guide more effective, individualised management strategies to attenuate reduced muscle health following hospitalisation.

## Conflicts of Interest

The authors declare no conflicts of interest.

## Supporting information


**Table S1** Risk of bias assessment results for observational cohort studies.
Table S2. Risk of bias assessment results for RCT studies.
Table S3. Summary of changes in HGS during hospitalisation.
Table S4. Summary of changes in KES during hospitalisation.
Table S5. Summary of whole‐body muscle mass changes during hospitalisation.
Table S6. Summary of muscle‐specific changes in muscle mass during hospitalisation.
Table S7. Summary of changes in muscle mass in the lower limbs during hospitalisation.
Table S8. Summary of changes in muscle function during hospitalisation.
Table S9. Summary of changes in muscle quality indices during hospitalisation.
