# Supplementary material for: Acute Sarcopenia: Systematic Review and Meta‐Analysis on Its Incidence and Muscle Parameter Shifts During Hospitalisation
Source: J Cachexia Sarcopenia Muscle. 2024 Dec 17;16(1):e13662. doi: 10.1002/jcsm.13662 (PMC11695106; doi:10.1002/jcsm.13662)
Supplement: Supplementary file 1 — Table S1 Risk of bias assessment results for observational cohort studies. Table S2. Risk of bias assessment results for RCT studies. Table S3. Summary of changes in HGS during hospitalisation. Table S4. Summary of changes in KES during hospitalisation. Table S5. Summary of whole‐body muscle mass changes during hospitalisation. Table S6. Summary of muscle‐specific changes in muscle mass during hospitalisation. Table S7. Summary of changes in muscle mass in the lower limbs during hospitalisation. Table S8. Summary of changes in muscle function during hospitalisation. Table S9. Summary of changes in muscle quality indices during hospitalisation. [file JCSM-16-e13662-s001.docx]

**Supplementary Material**

***Search terms used for all databases:***

((surgery OR bed rest OR immobilization OR "hospitalization" OR critically ill OR injury OR ICU) AND ("acute sarcopenia" OR sarcopenia OR "appendicular lean mass" OR "muscle index" OR "muscle cross-sectional area" OR "quadriceps cross-sectional area" OR handgrip strength OR "gait speed" OR "chair stand" OR "timed up and go" OR SPPB OR "6 minute walk*"))

Additional filters included:

- Humans
- Adults (18+ years)
- English language

***Table S1.*** *Risk of bias assessment results for observational cohort studies.*

| Observational Studies |  |  |  |  |  |  |  |  |  |  |  |  |  |  |  |  |  |  |  |  |  |  |  |  |  |  |  |  |  |  |  |
| --- | --- | --- | --- | --- | --- | --- | --- | --- | --- | --- | --- | --- | --- | --- | --- | --- | --- | --- | --- | --- | --- | --- | --- | --- | --- | --- | --- | --- | --- | --- | --- |
|  | LA | KP | LA | KP | LA | KP | LA | KP | LA | KP | LA | KP | LA | KP | LA | KP | LA | KP | LA | KP | LA | KP | LA | KP | LA | KP | LA | KP | LA | KP | **Rating** |
|  | **1** | | **2** | | **3** | | **4** | | **5** | | **6** | | **7** | | **8** | | **9** | | **10** | | **11** | | **12** | | **13** | | **14** | | **Score/14** | |  |
| Aarden et al. (2021) | + | + | + | + | + | + | + | + | - | - | + | + | + | + | - | - | NR | NR | + | + | + | + | - | - | + | + | + | + | 10 | 10 | Fair |
| Annetta et al. (2017) | + | + | + | + | + | + | + | + | - | - | + | + | + | + | + | + | NR | NR | + | + | + | + | - | - | - | - | + | + | 10 | 10 | Fair |
| Attaway et al. (2022) | + | + | + | + | + | + | + | + | + | + | + | + | + | + | + | + | NR | NR | + | + | + | + | - | - | + | + | - | - | 11 | 11 | Good |
| Ballasteros-Pomar et al. (2021) | + | + | + | + | - | - | + | + | + | + | + | + | + | + | + | + | NR | NR | + | + | + | + | - | - | + | + | - | - | 10 | 10 | Fair |
| Bodilsen et al. (2013) | + | + | + | + | - | - | + | + | + | + | + | + | + | + | - | - | NR | NR | - | - | + | + | CD | CD | - | - | - | - | 7 | 7 | Poor |
| Borges et al. (2020) | + | + | + | + | + | + | + | + | - | - | + | + | + | + | - | - | NR | NR | + | + | + | + | - | - | + | + | + | + | 10 | 10 | Fair |
| Bradford et al. (2023) | + | + | + | + | + | + | + | + | - | - | + | + | + | + | + | + | NR | NR | + | + | + | + | - | - | + | + | - | - | 10 | 10 | Fair |
| Butera et al. (2024) | + | + | + | + | CD | CD | - | - | - | - | + | + | + | + | - | + | NR | NR | - | - | + | + | - | - | + | + | - | - | 6 | 6 | Poor |
| Chites et al. (2021) | + | + | + | + | NR | NR | - | - | + | + | + | + | + | + | - | - | NR | NR | + | + | + | + | - | - | - | - | + | + | 8 | 8 | Poor |
| Da Silva et al. (2022) | + | + | + | + | + | + | + | + | - | - | + | + | + | + | + | + | NR | NR | + | + | - | - | - | - | + | + | - | - | 9 | 9 | Fair |
| De Andrade Junior et al. (2021) | + | + | + | + | + | + | + | + | - | - | + | + | + | + | - | - | NR | NR | + | + | + | + | - | - | - | - | - | - | 8 | 8 | Poor |
| de Buyser et al. (2014) | + | + | + | + | + | + | + | + | - | - | + | + | + | + | + | + | NR | NR | + | + | + | + | - | - | + | + | - | - | 10 | 10 | Fair |
| De Carvalho et al. (2022) | + | + | + | + | + | + | - | - | - | - | + | + | + | + | - | - | NR | NR | + | + | + | + | - | - | - | - | - | - | 7 | 7 | Poor |
| de Moura et al. (2023) | + | + | + | + | + | + | + | + | + | + | + | + | + | + | + | + | NR | NR | - | - | + | + | - | - | + | + | - | - | 10 | 10 | Fair |
| Dimopoulos et al. (2020) | + | + | + | + | + | + | + | + | - | - | + | + | + | + | + | + | NR | NR | + | + | + | + | - | - | - | - | - | - | 9 | 9 | Fair |
| Dusseaux et al. (2019) | + | + | + | + | + | + | - | - | - | - | + | + | + | + | - | - | NR | NR | + | + | + | + | + | + | + | + | - | - | 9 | 9 | Fair |
| Gil et al. (2022) | - | - | + | + | CD | CD | + | + | - | - | + | + | + | + | + | + | NR | NR | + | + | + | + | + | + | + | + | - | - | 9 | 9 | Fair |
| Gualtieri et al. (2020) | + | + | + | + | + | + | + | + | + | + | + | + | + | + | + | + | NR | NR | + | + | + | + | - | - | + | + | - | - | 11 | 11 | Good |
| Hadda et al. (2018) | + | + | + | + | + | + | + | + | + | + | + | + | + | + | + | + | NR | NR | + | + | + | + | - | - | - | - | - | - | 10 | 10 | Fair |
| Haines et al. (2019) | - | - | + | + | + | + | + | + | - | - | + | + | + | + | + | + | NR | NR | + | + | - | - | + | + | + | + | - | - | 9 | 9 | Fair |
| Hayes et al. (2018) | + | + | + | + | + | + | + | + | + | + | + | + | + | + | + | + | NR | NR | + | + | + | + | - | - | - | - | - | - | 10 | 10 | Fair |
| Kangalgil et al. (2022) | + | + | + | + | + | + | - | - | - | - | + | + | + | + | + | + | NR | NR | + | + | + | + | - | - | + | + | - | - | 9 | 9 | Fair |
| Kangalgil et al. (2024) | + | + | + | + | + | + | - | - | + | + | + | + | + | + | + | + | NR | NR | + | + | + | + | - | - | + | + | + | + | 11 | 11 | Good |
| Karlsen et al. (2017) | + | + | + | + | + | + | - | - | + | + | + | + | + | + | - | - | NR | NR | + | + | + | + | - | - | - | - | - | - | 8 | 8 | Poor |
| Katari et al. (2018) | + | + | + | + | NR | NR | + | + | - | - | + | + | + | + | + | + | NR | NR | + | + | + | + | - | - | CD | CD | - | - | 8 | 8 | Poor |
| Kim et al. (2023) | + | + | + | + | + | + | + | + | + | + | + | + | + | + | - | - | + | + | + | + | + | + | - | - | + | + | - | - | 11 | 11 | Good |
| Kouw et al. (2019) | + | + | + | + | + | + | + | + | + | + | + | + | + | + | - | - | + | + | - | - | + | + | + | + | - | - | NR | NR | 10 | 10 | Fair |
| Lee et al. (2021) | + | + | + | + | + | + | + | + | - | - | + | + | + | + | + | + | NR | NR | + | + | - | - | - | - | + | + | + | + | 10 | 11 | Good |
| Lopez Jimenez et al. (2024) | + | + | + | + | + | + | - | - | - | - | + | + | + | + | - | - | NR | NR | + | + | - | + | - | - | + | + | - | - | 7 | 7 | Poor |
| Martín-Salvador et al. (2015) | + | + | + | + | + | + | + | + | - |  | + | + | + | + | - | - | + | + | + | + | + | + | - | - | + | + | - | - | 10 | 10 | Fair |
| Martone et al. (2017) | + | + | + | + | + | + | + | + | - | - | + | + | + | + | - | - | NR | NR | + | + | + | + | - | - | + | + | + | + | 10 | 10 | Fair |
| Mayer et al. (2020) | + | + | + | + | + | + | + | + | - | - | + | + | + | + | + | + | NR | NR | + | + | + | + | - | - | + | + | + | + | 11 | 11 | Good |
| Mgbemena et al. (2022) | + | + | + | + | + | + | + | + | + | + | + | + | + | + | + |  | - | - | + | + | + | + | NR | NR | + | + | - | - | 12 | 12 | Good |
| Nakanishi et al. (2020) | + | + | + | + | + | + | + | + | - | - | + | + | + | + | + | + | NR | NR | + | + | + | + | + | + | + | + | - | - | 11 | 11 | Good |
| Norheim et al. (2017) | + | + | + | + | + | + | + | + | - | - | + | + | + | + | - | - | NR | NR | - | - | + | + | - | - | - | - | - | - | 7 | 7 | Poor |
| Parry et al. (2015) | + | + | - | - | NR | NR | + | + | - | - | + | + | + | + | + | + | NR | NR | + | + | + | + | - | - | CD | CD | - | - | 8 | 8 | Poor |
| Pitta et al. (2006) | + | + | + | + | + | + | + | + | + | + | + | + | + | + | + | + | NR | NR | + | + | + | + | - | - | - | - | - | - | 10 | 10 | Fair |
| Pourhassan et al. (2020) | + | + | + | + | NR | NR | + | + | + | + | + | + | + | + | + | + | NR | NR | + | + | + | + | + | + | + | + | + | + | 12 | 12 | Good |
| Puthucheary et al. (2013) | + | + | + | + | + | + | + | + | + | + | + | + | + | + | + | + | NR | NR | + | + | + | + | + | + | + | + | + | + | 13 | 13 | Good |
| Ramsey et al. (2022) | + | + | + | + | + | + | + | + | - | - | + | + | + | + | + | + | NR | NR | + | + | + | + | - | - | + | + | - | - | 10 | 10 | Fair |
| Rodrigues et al. (2021) | + | + | + | + | + | + | + | + | + | + | + | + | + | + | + | + | NR | NR | + | + | + | + | - | - | - | - | + | + | 11 | 11 | Good |
| Segaran et al. (2017) | + | + | + | + | - | - | - | - | - | - | + | + | + | + | + | + | NR | NR | + | + | + | + | - | - | - | - | - | - | 7 | 7 | Poor |
| Tazerout et al. (2022) | + | + | - | - | + | + | + | + | - | - | + | + | + | + | + | + | NR | NR | CD | CD | + | + | + | + | + | + | + | + | 10 | 10 | Fair |
| Toledo et al. (2021) | + | + | + | + | + | + | + | + | - | - | + | + | + | + | + | + | NR | NR | + | + | + | + | - | - | + | + | - | - | 10 | 10 | Fair |
| Trung et al. (2019) | + | + | + | + | NR | NR | + | + | + | + | + | + | + | + | - | - | NR | NR | + | + | + | + | - | - | + | + | - | - | 9 | 9 | Fair |
| Turton et al. (2016) | + | + | + | + | + | + | + | + | - | - | + | + | + | + | - | - | NR | NR | + | + | + | + | - | - | - | - | - | - | 8 | 8 | Poor |
| Welch et al. (2022a) | + | + | + | + | NR | NR | - | - | + | + | + | + | + | + | + | + | - | - | + | + | + | + | - | - | - | - | NR | NR | 8 | 8 | Poor |
| Welch et al. (2022b) | + | + | + | + | NR | NR | - | - | + | + | + | + | + | + | + | + | - | - | + | + | + | + | - | - | - | - | NR | NR | 8 | 8 | Poor |
| Werner et al. (2024) | + | + | + | + | - | - | + | + | + | + | + | + | + | + | + | + | NR | NR | + | + | + | + | - | - | - | - | + | + | 10 | 10 | Fair |
| Xie et al. (2019) | + | + | + | + | + | + | + | + | - | - | + | + | + | + | - | - | NR | NR | + | + | + | + | - | - | - | - | - | - | 8 | 8 | Poor |
| 1. Was the research question or objective in this paper clearly stated? 2. Was the study population clearly specified and defined? 3. Was the participation rate of eligible persons at least 50%? 4. Were all the subjects selected or recruited from the same or similar populations (including the same time period)? Were inclusion and exclusion criteria for being in the study prespecified and applied uniformly to all participants? 5. Was a sample size justification, power description, or variance and effect estimates provided? 6. For the analyses in this paper, were the exposure(s) of interest measured prior to the outcome(s) being measured? 7. Was the timeframe sufficient so that one could reasonably expect to see an association between exposure and outcome if it existed? 8. For exposures that can vary in amount or level, did the study examine different levels of the exposure as related to the outcome (e.g., categories of exposure, or exposure measured as continuous variable)? 9. Were the exposure measures (independent variables) clearly defined, valid, reliable and implemented consistently across all participants? 10. Was the exposure(s) assessed more than once over time? 11. Were the outcome measures (dependent variables) clearly defined, valid, reliable and implemented consistently across all study participants? 12. Were the outcome assessors blinded to the exposure status of the participants? 13. Was loss to follow-up after baseline 20% or less? 14. Were key potential confounding variables measured and adjusted statistically for their impact on the relationship between exposure(s) and outcome(s)? N/A = Not Applicable; NR = Not Reported; CD = Cannot Determine. | | | | | | | | | | | | | | | | | | | | | | | | | | | | | | | |

***Table S2.*** *Risk of bias assessment results for RCT studies.*

| RCTs |  |  |  |  |  |  |  |  |  |  |  |  |  |  |  |  |  |  |  |  |  |  |  |  |  |  |  |  |  |  |  |  |
| --- | --- | --- | --- | --- | --- | --- | --- | --- | --- | --- | --- | --- | --- | --- | --- | --- | --- | --- | --- | --- | --- | --- | --- | --- | --- | --- | --- | --- | --- | --- | --- | --- |
| Ahmad et al. (2024) | + | + | + | + | + | + | + | + | - | - | - | - | + | + | + | + | NR | NR | + | + | + | + | + | + | + | + | + | + | 11 | 11 | Good |  |
| Bao et al. (2022) | + | + | + | + | + | + | + | + | + | + | + | + | + | + | + | + | + | + | + | + | + | + | + | + | + | + | - | - | 12 | 12 | Good |  |
| Barbalho et al. (2019) | + | + | - | - | NR | NR | - | - | + | + | + | + | + | + | + | + | + | + | + | + | + | + | - | - | + | + | NR | NR | 9 | 9 | Fair |  |
| Beyer et al. (2011) | + | + | + | + | NR | NR | + | + | + | + | - | - | - | - | - | - | - | - | CD | CD | + | + | + | + | + | + | NR | NR | 7 | 7 | Poor |  |
| Bologna & Pone (2022) | + | + | NR | NR | NR | NR | NR | NR | NR | NR | - | - | + | + | + | + | CD | CD | + | + | + | + | - | - | + | + | NR | NR | 6 | 6 | Poor |  |
| Borges et al. (2016) | + | + | + | + | CD | CD | - | - | - | - | + | + | + | + | + | + | NR | NR | + | + | + | + | - | - | + | + | NR | NR | 8 | 8 | Poor |  |
| Chapple et al. (2022) | + | + | NR | NR | NR | N | + | + | + | + | + | + | - | - | + | + | + | + | + | + | + | + | - | - | + | + | - | - | 9 | 9 | Fair |  |
| de Morton et al. (2007) | + | + | + | + | + | + | - | - | - | - | + | + | - | - | + | + | + | + | + | + | + | + | + | + | + | + | + | + | 11 | 11 | Good |  |
| Dirks et al. (2015) | + | + | + | + | + | + | - | - | + | + | + | + | - | - | + | + | + | + | + | + | + | + | + | + | + | + | NR | NR | 11 | 11 | Good |  |
| Eibel et al. (2022) | + | + | + | + | + | + | - | - | NR | NR | + | + | + | + | + | + | NR | NR | + | + | + | + | + | + | + | + | + | + | 11 | 11 | Good |  |
| Fränzel et al. (2024) | + | + | + | + | + | + | + | + | - | - | + | + | + | + | + | + | + | + | + | + | + | + | + | + | + | + | - | - | 12 | 12 | Good |  |
| Gerovasili et al. (2009) | + | + | CD | CD | + | + | - | - | + | + | + | + | - | - | - | - | - | - | + | + | + | + | - | - | + | + | NR | NR | 7 | 7 | Poor |  |
| Giangregorio et al. (2009) | - | - | NA | - | NA | - | - | - | - | - | + | + | - | - | - | - | - | - | + | + | + | + | - | - | + | + | - | - | 4 | 4 | Poor |  |
| Hasanloei et al. (2021a) | + | + | + | + | + | + | + | + | + | + | + | + | + | + | + | + | + | + | + | + | + | + | + | + | + | + | NR | NR | 13 | 13 | Good |  |
| Hasanloei et al. (2021b) | + | + | + | + | + | + | + | + | + | + | + | + | + | + | + | + | + | + | + | + | + | + | + | + | + | + | NR | NR | 13 | 13 | Good |  |
| Hickson et al. (2004) | + | + | + | + | + | + | - | - | - | - | + | + | + | + | + | + | NR | NR | - | - | + | + | - | - | + | + | + | + | 9 | 9 | Fair |  |
| Hu et al. (2020) | + | + | - | - | + | + | - | - | NR | NR | + | + | - | - | - | - | - | - | + | + | + | + | - | - | + | + | NR | NR | 6 | 6 | Poor |  |
| Jones et al. (2006) | + | + | + | + | + | + | + | + | CD | CD | - | - | + | + | + | + | + | + | + | + | + | + | + | + | + | + | + | + | 12 | 12 | Good |  |
| Kronborg et al. (2017) | + | + | + | + | + | + | + | + | + | + | + | + | - | - | + | + | + | + | + | + | + | + | + | + | + | + | + | + | 13 | 13 | Good |  |
| Martínez-Velila et al. (2019) | + | + | NR | NR | + | + | - | - | + | + | + | + | - | - | + | + | + | + | + | + | + | + | NR | NR | + | + | + | + | 10 | 10 | Good |  |
| McNelly et al. (2020) | + | + | + | + | + | + | + | + | + | + | + | + | - | - | + | + | + | + | + | + | + | + | + | + | + | + | + | + | 13 | 13 | Fair |  |
| Mets et al. (2004) | + | + | CD | CD | NR | NR | - | - | + | + | + | + | + | + | + | + | + | + | + | + | + | + | + | + | + | + | NR | NR | 10 | 10 | Fair |  |
| Nery et al. (2012) | - | - | - | - | + | + | - | - | + | + | + | + | + | + | + | + | NR | NR | + | + | + | + | - | - | + | + | NR | NR | 8 | 8 | Good |  |
| Neto et al. (2024) | + | + | NR | NR | CD | CD | - | - | + | + | + | + | + | + | + | + | CD | CD | + | + | + | + | + | + | + | + | + | + | 10 | 10 | Fair |  |
| Nickels et al. (2020) | + | + | + | + | + | + | - | - | + | + | + | + | + | + | + | + | - | - | + | + | + | + | + | + | + | + | + | + | 12 | 12 | Good |  |
| Ogasawara et al. (2018) | + | + | + | + | + | + | NR | NR | NR | NR | + | + | + | + | + | + | NR | NR | - | - | + | + | + | + | + | + | - | - | 9 | 9 | Fair |  |
| Ortiz-Alonso et al. (2019) | + | + | - | - | + | + | - | - | - | - | + | + | + | + | + | + | NR | NR | + | + | + | + | - | - | + | + | + | + | 9 | 9 | Fair |  |
| Raymond et al. (2017) | + | + | + | + | + | + | + | + | - | - | + | + | + | + | + | + | CD | CD | + | + | + | + | + | + | + | + | + | + | 12 | 12 | Good |  |
| Segers et al. (2021) | + | + | + | + | - | - | - | - | + | + | + | + | + | + | + | + | + | + | + | + | + | + | + | + | + | + | NR | NR | 11 | 11 | Good |  |
| Silva et al. (2019) | + | + | + | + | + | + | - | - | + | + | + | + | - | - | + | + | + | + | + | + | + | + | + | + | + | + | + | + | 12 | 12 | Good |  |
| Strasser et al. (2023) | + | + | + | + | + | + | - | - | - | - | + | + | + | + | + | + | + | + | + | + | + | + | - | - | + | + | + | + | 11 | 11 | Good |  |
| Temporiti et al. (2022) | + | + | + | + | + | + | + | + | + | + | + | + | + | + | + | + | CD | CD | + | + | + | + | + | + | + | + | - | - | 12 | 12 | Good |  |
| Torres-Sámchez et al. (2017) | + | + | + | + | + | + | + | + | + | + | + | + | + | + | + | + | NR | NR | + | + | + | + | + | + | + | + | + | + | 13 | 13 | Good |  |
| Verceles et al. (2023) | + | + | + | + | - | - | - | - | - | - | + | + | - | - | + | + | - | - | + | + | + | + | - | CD | + | + | - | - | 7 | 7 | Poor |  |
| Vermeeren et al. (2004) | + | + | - | - | NR | NR | + | + | NR | NR | + | + | + | + | NR | NR | NR | NR | + | + | + | + | - | - | + | + | - | - | 7 | 7 | Poor |  |
| Wnuk et al. (2016) | + | + | - | - | + | + | - | - | - | - | + | + | + | + | + | + | NR | NR | + | + | + | + | - | - | + | + | NR | NR | 8 | 8 | Poor |  |
| Wu et al. (2023) | + | + | + | + | + | + | + | + | + | + | + | + | + | + | + | + | + | + | + | + | + | + | + | + | + | + | + | + | 14 | 14 | Good |  |
| Xue et al. (2022) | + | + | + | + | + | + | + | + | - | - | + | + | + | + | + | + | + | + | + | + | + | + | + | + | + | + | + | + | 13 | 13 | Good |  |
| 1. Was the study described as randomized , a randomized trial a randomized clinical trial or an RCT? 2. Was the method of randomization adequate (i.e., use of randomly generated assignment)? 3. Was the treatment allocation concealed (so that assignments could not be predicted)? 4. Were study participants and providers blinded to treatment group assignment? 5. Were the people assessing the outcomes blinded to the participants' group assignments? 6. Were the groups similar at baseline on important characteristics that could affect outcomes (e.g., demographics, risk factors, co-morbid conditions)? 7. Was the overall drop-out rate from the study at endpoint 20% or lower of the number allocated to treatment? 8. Was the differential drop-out rate (between treatment groups) at endpoint 15 percentage points or lower? 9. Was there high adherence to the intervention protocols for each treatment group? 10. Were other interventions avoided or similar in the groups (e.g., similar background treatments)? 11. Were outcomes assessed using valid and reliable measures, implemented consistently across all study participants? 12. Did the authors report that the sample size was sufficiently large to be able to detect a difference with the main outcome between groups with at least 80% power? 13. Were outcomes reported or subgroups analyzed prespecified (i.e., identified before analyses were conducted)? 14. Were all randomized participants analyzed in the group to which they were originally assigned i.e., did they use an intention-to-treat analysis? N/A = Not Applicable; NR = Not Reported; CD = Cannot Determine. | | | | | | | | | | | | | | | | | | | | | | | | | | | | | | | |  |
|  |  |  |  |  |  |  |  |  |  |  |  |  |  |  |  |  |  |  |  |  |  |  |  |  |  |  |  |  |  |  |  |  |

***Table S3****. Summary of changes in HGS during hospitalisation.*

| **Author(s)** | **Population** | **Exposure** | **Sex** | **Number** | **Measurement Tool** | **Outcome on HGS** | **Duration (days)** | ***p* Value** |
| --- | --- | --- | --- | --- | --- | --- | --- | --- |
| Aarden et al. (2021)* | Various | Medical ward | M | 174 | JAMAR Dynamometer | ↗ | 6 | NR |
| Aarden et al. (2021)* | Various | Medical ward | F | 169 | JAMAR Dynamometer | ↗ | 6 | NR |
| Beyer et al. (2011) | Acute infection | Medical ward | M/F | 14 | Martin Vigorometer | ↔ | 21 | NR |
| Bodilsen et al. (2013)* | Various | Medical ward | M | 12 | Digi-II Dynamometer | ↙ | 7.5 | NR |
| Bodilsen et al. (2013)* | Various | Medical ward | F | 11 | Digi-II Dynamometer | ↙ | 7.5 | NR |
| Bologna & Pone (2022) | COVID-19 | ICU | M/F | 40 | JAMAR Dynamometer | ↓ | 20 | NR |
| Borges et al. (2020)* | Sepsis | Surgery | M/F | 45 | Takei Dynamometer | ↗ | 20 | NR |
| Chites et al. (2021)* | Various | Medical ward, Surgery & ICU | M | 65 | Digi-II Dynamometer | ↗ | 14 | NR |
| Chites et al. (2021)* | Various | Medical ward, Surgery & ICU | F | 69 | Digi-II Dynamometer | ↗ | 14 | NR |
| Da Silva et al. (2022) | Burns | ICU & Medical Ward | M/F | 41 | JAMAR Dynamometer | ↓, ↗ | 30 | <0.05 |
| De Andrade-Juníor et al. (2021) | COVID-19 | ICU | M/F | 9 | JAMAR Dynamometer | ↓ | 10 | <0.05 |
| De Buyser et al. (2014) | Various | Medical ward | M/F | 639 | North Coast Medical Dynamometer | ↑ | 9 | 0.001 |
| De Carvalho et al. (2022) | Various | Medical ward & Surgery | M | 679 | JAMAR Dynamometer | ↓ | 8 | <0.01 |
| De Carvalho et al. (2022) | Various | Medical ward & Surgery | F | 489 | JAMAR Dynamometer | ↓ | 7 | <0.01 |
| Gil et al. (2022) | COVID-19 | Medical ward & ICU | M/F | 80 | Takei Dynamometer | ↓ | 8 | NR |
| Hickson et al. (2004) | Various | Medical ward | M/F | 300 | NR | ↗ | 23 | NR |
| Hu et al. (2020)* | Various | Medical ward | M | 25 | JAMAR Dynamometer | ↗ | 7 | NR |
| Hu et al. (2020)* | Various | Medical ward | F | 25 | JAMAR Dynamometer | ↗ | 7 | NR |
| Karlsen et al. (2017)* | Various | Medical ward | M/F | 151 | JAMAR Dynamometer | ↙ | 9 - 13 | NR |
| Kim et al. (2023)* | Hip disease | Medical ward | M/F | 57 | Takei Dynamometer | ↗ | 9 | >0.999 |
| Kim et al. (2023)* | Hip fracture | Surgery | M/F | 58 | Takei Dynamometer | ↑ | 10 | 0.015 |
| Kouw et al. (2019) | Osteoarthritis | Surgery | M/F | 26 | JAMAR Dynamometer | ↔ | 6 | >0.05 |
| Martínez-Velilla et al. (2019) | Various | Medical ward & Surgery | M/F | 185 | NR | ↙ | 8 | NR |
| Martín-Salvador et al. (2015) | Pneumonia (<75 years old, dominant hand) | Medical ward | M/F | 68 | TEC-60 Dynamometer | ↗ | 8 | 0.362 |
| Martín-Salvador et al. (2015) | Pneumonia (<75 years old, non-dominant hand) | Medical ward | M/F | 68 | TEC-60 Dynamometer | ↑ | 8 | <0.001 |
| Martín-Salvador et al. (2015) | Pneumonia (>75 years old, dominant hand) | Medical ward | M/F | 48 | TEC-60 Dynamometer | ↓ | 8 | 0.001 |
| Martín-Salvador et al. (2015) | Pneumonia (>75 years old, non-dominant hand) | Medical ward | M/F | 48 | TEC-60 Dynamometer | ↓ | 8 | 0.001 |
| Mets et al. (2004) | Acute infection | Medical ward | M/F | 15 | Martin Vigorometer | ↔ | 14 | NR |
| Mgbemena et al. (2022)* | Cardiac Surgery | ICU | M/F | 101 | JAMAR Dynamometer | ↓ | 11 | <0.001 |
| Neto et al. (2024)* | COVID-19 | ICU | M/F | 30 | NR | ↙ | 5 | 0.052 |
| Norheim et al. (2017) | Various - Reducing inflammation | Medical ward | M/F | 75 | JAMAR Dynamometer | ↗ | 6 | <0.001 |
| Norheim et al. (2017) | Various Continual Inflammation | Medical ward | M/F | 132 | JAMAR Dynamometer | ↔ | 7 | 0.707 |
| Pourhassan et al. (2020)* | Various - Malnourished | Medical ward | M | 3 | JAMAR Dynamometer | ↔ | 11.5 | NR |
| Pourhassan et al. (2020)* | Various - non-malnourished | Medical ward | M | 8 | JAMAR Dynamometer | ↙ | 13.7 | NR |
| Pourhassan et al. (2020)* | Various - Malnourished | Medical ward | F | 4 | JAMAR Dynamometer | ↗ | 12.5 | NR |
| Pourhassan et al. (2020)* | Various - non-malnourished | Medical ward | F | 26 | JAMAR Dynamometer | ↔ | 13.6 | NR |
| Ramsey et al. (2022)* | Various | Medical ward | F | 275 | NR | ↗ | 19.8 | 0.198 |
| Ramsey et al. (2022)* | Various | Medical ward | M | 199 | NR | ↑ | 19.8 | 0.001 |
| Strasser et al. (2023)* | Various | Medical ward | M/F | 10 | SAEHAN Dynamometer | ↗ | 21 | 0.444 |
| Vermeeren et al. (2004) | COPD | Medical ward | M/F | 24 | NR | ↔ | 8 | NR |
| Welch et al. (2022)* | Various | Medical wards & Surgery | M | 48 | JAMAR Dynamometer | ↙ | 7 | NR |
| Welch et al. (2022)* | Various | Medical wards & Surgery | F | 31 | JAMAR Dynamometer | ↙ | 7 | NR |
| Werner et al. (2024) | Various | Medical ward | M/F | 107 | NR | ↔ | 20 | 0.207 |
| Wu et al. (2023)* | Various | ICU | M/F | 28 | NR | ↗ | 13 | NR |
| COPD = Chronic Obstructive Pulmonary Disease; ICU = Intensive Care Unit; Sex: M = Male, F = Female; NR = Not reported; * = included in meta-analysis. ↓ = significant reduction; ↙ = non-significant reduction; ↔ = no change; ↗ = non-significant increase; ↑ = significant increase. | | | | | | | | |

***Table S4.*** *Summary of changes in KES during hospitalisation*

| **Author(s)** | **Population** | **Exposure** | **Sex** | **Number** | **Measurement Tool** | **Outcome on KES** | **Duration (days)** | ***p* Value** |
| --- | --- | --- | --- | --- | --- | --- | --- | --- |
| Bodilsen et al. (2013) | Various | Medical ward | M/F | 33 | Power Track II Commander | ↗ | 7.5 | 0.138 |
| Kronborg et al. (2017) | Hip fracture | Surgery | M/F | 45 | Power Track II Commander | ↗ | 10 | NR |
| Martín-Salvador et al. (2015) | Pneumonia (<75 years old, right quadriceps) | Medical ward | M/F | 68 | NR | ↓ | 8 | 0.04 |
| Martín-Salvador et al. (2015) | Pneumonia (<75 years old, left quadriceps) | Medical ward | M/F | 68 | NR | ↓ | 8 | 0.014 |
| Martín-Salvador et al. (2015) | Pneumonia (>75 years old, right quadriceps) | Medical ward | M/F | 48 | NR | ↓ | 8 | 0.009 |
| Martín-Salvador et al. (2015) | Pneumonia (>75 years old, left quadriceps) | Medical ward | M/F | 48 | NR | ↓ | 8 | 0.011 |
| Pitta et al. (2006) | COPD | Medical ward | M/F | 17 | NR | ↓ | 8 | <0.05 |
| Pourhassan et al. (2020) | Various - NOT malnourished | Medical ward | M/F | 34 | Strength Measuring Device FK | ↙ | 13 | NR |
| Pourhassan et al. (2020) | Various - malnourished | Medical ward | M/F | 7 | Strength Measuring Device FK | ↓ | 14 | NR |
| Segers et al. (2021) | Various | ICU | M/F | 12 | CompuFet 2 Dynamometer | ↔ | 7 | NR |
| Torres-Sánchez et al. (2017) | COPD | Medical ward | M/F | 29 | NR | ↙ | 10 | NR |
| Vermeeren et al. (2004) | COPD | Medical ward | M/F | 24 | NR | ↗ | 8 | NR |
| COPD = Chronic Obstructive Pulmonary Disease; ICU = Intensive Care Unit; Sex: M = Male, F = Female; NR = Not reported. ↓ = significant reduction; ↙ = non-significant reduction; ↔ = no change; ↗ = non-significant increase; ↑ = significant increase. | | | | | | | | |

***Table S5.*** *Summary of whole-body muscle mass changes during hospitalisation.*

| **Author(s)** | **Population** | **Exposure** | **Sex** | **Number** | **Measurement Tool** | **Outcome on Muscle Mass** | **Duration (days)** | ***p* Value** |
| --- | --- | --- | --- | --- | --- | --- | --- | --- |
| Aarden et al. (2021) | Various | Medical ward | M/F | 343 | BIA | ↔ | 6 | NR |
| Beyer et al. (2011) | Acute Infection | Medical ward | M/F | 14 | Isotopic Potassium | ↔ | 21 | NR |
| Hasanloei et al. (2021a) | Various | ICU | M/F | 50 | BIA | ↓ | 36 | <0.001 |
| Hasanloei et al. (2021b) | Trauma | ICU | M/F | 20 | BIA | ↙ | 7 | 0.05 |
| Ogasawara et al. (2018) | COPD (LBMI) | Medical ward | M/F | 21 | BIA | ↗ | 12 | 0.36 |
| Ogasawara et al. (2018) | COPD (SMI) | Medical ward | M/F | 21 | BIA | ↙ | 12 | 0.35 |
| Pitta et al. (2006) | COPD | Medical Ward | M/F | 17 | BIA | ↔ | 8 | NR |
| Strasser et al. (2023) | Various | Medical ward | M/F | 10 | NR | ↗ | 7 | 0.647 |
| Vermeeren et al. (2004) | COPD | Medical ward | M/F | 24 | BIA | ↙ | 8 | NR |
| Welch et al. (2022a) | Colorectal surgery & Acute Infection | Medical ward & surgery | M | 48 | BIA | ↓ | 7 | NR |
| Welch et al. (2022a) | Colorectal surgery & Acute Infection | Medical ward & surgery | F | 31 | BIA | ↓ | 7 | NR |
| COPD = Chronic Obstructive Pulmonary Disease; ICU = Intensive Care Unit; BIA = Bio-electrical impedance analysis; LBMI = Lean body mass index; SMI = skeletal muscle index; Sex: M = Male, F = Female; NR = Not reported. ↓ = significant reduction; ↙ = non-significant reduction; ↔ = no change; ↗ = non-significant increase; ↑ = significant increase. | | | | | | | | |

***Table S6.*** *Summary of muscle-specific changes in muscle mass during hospitalisation.*

| **Author(s)** | **Population** | **Exposure** | **Sex** | **Number** | **Measurement Tool** | **Muscle Assessed** | **Outcome on Muscle Mass** | **Duration (days)** | ***p* Value** |
| --- | --- | --- | --- | --- | --- | --- | --- | --- | --- |
| Attaway et al. (2022) | COVID-19 | Medical ward & ICU | M/F | 95 | CT | Erector Spinae | ↓ | 30 | 0.003 |
| Attaway et al. (2022) | COVID-19 | Medical ward & ICU | M/F | 95 | CT | Pectoralis | ↓ | 30 | <0.001 |
| Bradford et al. (2023) | Trauma | ICU | M/F | 81 | CT | Psoas | ↓ | 38 | <0.001 |
| Chapple et al. (2022) | Various | ICU | M/F | 36 | Ultrasound | Forearm | ↙ | <60 | NR |
| Chapple et al. (2022) | Various | ICU | M/F | 39 | Ultrasound | Mid-upper arm | ↙ | <60 | NR |
| De Moura et al. (2023) | COVID-19 | ICU | M/F | 30 | Ultrasound | Deltoid (right) | ↓ | 7 | 0.004 |
| De Moura et al. (2023) | COVID-19 | ICU | M/F | 30 | Ultrasound | Biceps Brachialis (right) | ↓ | 7 | 0.002 |
| De Moura et al. (2023) | COVID-19 | ICU | M/F | 30 | Ultrasound | Deltoid (left) | ↓ | 7 | 0.005 |
| De Moura et al. (2023) | COVID-19 | ICU | M/F | 30 | Ultrasound | Biceps Brachialis (left) | ↓ | 7 | <0.001 |
| De Moura et al. (2023) | COVID-19 | ICU | M/F | 30 | Ultrasound | Rectus abdominus | ↓ | 7 | 0.003 |
| De Moura et al. (2023) | COVID-19 | ICU | M/F | 30 | Ultrasound | Transverse Abdominus | ↓ | 7 | 0.024 |
| De Moura et al. (2023) | COVID-19 | ICU | M/F | 30 | Ultrasound | Internal Oblique | ↓ | 7 | 0.001 |
| De Moura et al. (2023) | COVID-19 | ICU | M/F | 30 | Ultrasound | External Oblique | ↙ | 7 | 0.096 |
| Dusseaux et al. (2019) | Various | Medical ward & ICU | M/F | 25 | CT | Abdomen | ↙ | 11 | 0.183 |
| Gualtieri et al. (2020) | COVID-19 | ICU | M/F | 30 | CT | Erector Spinae | ↓ | 20 | <0.001 |
| Gualtieri et al. (2020) | COVID-19 (Obese) | ICU | M/F | 17 | CT | Erector Spinae | ↓ | 20 | <0.001 |
| Gualtieri et al. (2020) | COVID-19 (Lean) | ICU | M/F | 13 | CT | Erector Spinae | ↙ | 20 | 0.09 |
| Hadda et al. (2018) | Sepsis | ICU | M/F | 70 | Ultrasound | Arm | ↓ | 7 | 0.013 |
| Haines et al. (2019) | Trauma (Group 1) | ICU | M/F | 24 | CT | Abdomen | ↔ | 6 | 0.08 |
| Haines et al. (2019) | Trauma (Group 2) | ICU | M/F | 25 | CT | Abdomen | ↔ | 4 | 0.2 |
| Haines et al. (2019) | Trauma (Group 3) | ICU | M/F | 19 | CT | Abdomen | ↓ | 16 | <0.001 |
| Haines et al. (2019) | Trauma (Group 4) | ICU | M/F | 21 | CT | Abdomen | ↓ | 44 | <0.001 |
| Haines et al. (2019) | Trauma (Group 1) | ICU | M/F | 24 | CT | L4 Psoas | ↓ | 6 | <0.001 |
| Haines et al. (2019) | Trauma (Group 2) | ICU | M/F | 25 | CT | L4 Psoas | ↓ | 4 | 0.016 |
| Haines et al. (2019) | Trauma (Group 3) | ICU | M/F | 19 | CT | L4 Psoas | ↓ | 16 | <0.001 |
| Haines et al. (2019) | Trauma (Group 4) | ICU | M/F | 21 | CT | L4 Psoas | ↓ | 44 | <0.001 |
| Nakanishi et al. (2020) | Various | ICU | M/F | 64 | Ultrasound | Biceps Brachii | ↓ | 7 | NR |
| Rodrigues et al. (2019) | Various (Dominant hand) | Medical & surgical ICU | M/F | 60 | Ultrasound | Adductor pollicis | ↓ | 7 | 0.007 |
| Segaran et al. (2017) | Various (Normal weight) | Medical & surgical ICU | M/F | 17 | Ultrasound | Biceps Brachii, forearm, thigh | ↓ | 12 | NR |
| Segaran et al. (2017) | Various (Overweight) | Medical & surgical ICU | M/F | 10 | Ultrasound | Biceps Brachii, forearm, thigh | ↓ | 12 | NR |
| Segaran et al. (2017) | Various (Obese) | Medical & surgical ICU | M/F | 17 | Ultrasound | Biceps Brachii, forearm, thigh | ↓ | 12 | NR |
| Tazerout et al. (2022) | Trauma | ICU | M/F | 114 | CT | Psoas | ↓ | 8 | NR |
| COPD = Chronic Obstructive Pulmonary Disease; ICU = Intensive Care Unit; CT = Computed tomography; Sex: M = Male, F = Female; NR = Not reported. ↓ = significant reduction; ↙ = non-significant reduction; ↔ = no change; ↗ = non-significant increase; ↑ = significant increase. | | | | | | | | | |

***Table S7.*** *Summary of changes in muscle mass in the lower limbs during hospitalisation.*

| **Author(s)** | **Population** | **Exposure** | **Sex** | **Number** | **Measurement Tool** | **Muscle Assessed** | **Outcome Measure** | **Outcome on Muscle Mass** | **Duration (days)** | ***p* Value** |
| --- | --- | --- | --- | --- | --- | --- | --- | --- | --- | --- |
| Annetta et al. (2017) | Trauma | ICU | M/F | 20 | Ultrasound | Rectus Femoris | CSA | ↓ | 20 | <0.05 |
| Annetta et al. (2017) | Trauma | ICU | M/F | 20 | Ultrasound | Anterior Tibialis | CSA | ↙ | 20 | 0.3 |
| Bao et al. (2022) | Various | ICU | M/F | 20 | CT | Lower leg | CSA | ↓ | 12 | <0.05 |
| Barbalho et al. (2019) | Various | ICU | M/F | 20 | Ultrasound | Quadriceps | Thickness | ↓ | 11 | <0.001 |
| Bologna & Pone (2022) | COVID-19 | ICU | M/F | 40 | Ultrasound | Vastus Lateralis | Thickness | ↓ | 20 | NR |
| Borges et al. (2020)* | Sepsis | ICU | M/F | 45 | Ultrasound | Rectus Femoris | CSA | ↓ | 20 | 0.001 |
| Chapple et al. (2022) | Various | ICU | M/F | 42 | Ultrasound | Quadriceps | Thickness | ↙ | <60 | NR |
| De Andrade-Juníor et al. (2021) | COVID-19 | ICU | M/F | 32 | Ultrasound | Rectus Femoris | CSA | ↓ | 10 | <0.05 |
| De Moura et al. (2023)* | COVID-19 | ICU | M/F | 13 | Ultrasound | Rectus Femoris (left) | CSA | ↙ | 7 | NR |
| De Moura et al. (2023)* | COVID-19 | ICU | M/F | 10 | Ultrasound | Rectus Femoris (right) | CSA | ↙ | 7 | NR |
| De Moura et al. (2023) | COVID-19 | ICU | M/F | 30 | Ultrasound | Quadriceps (right) | Thickness | ↓ | 7 | <0.001 |
| De Moura et al. (2023) | COVID-19 | ICU | M/F | 30 | Ultrasound | Rectus Femoris (right) | Thickness | ↓ | 7 | 0.001 |
| De Moura et al. (2023) | COVID-19 | ICU | M/F | 30 | Ultrasound | Vastus Intermedius (right) | Thickness | ↓ | 7 | 0.002 |
| De Moura et al. (2023) | COVID-19 | ICU | M/F | 30 | Ultrasound | Medial Gastrocnemius (right) | Thickness | ↓ | 7 | <0.001 |
| De Moura et al. (2023) | COVID-19 | ICU | M/F | 30 | Ultrasound | Lateral Gastrocnemius (right) | Thickness | ↓ | 7 | <0.001 |
| De Moura et al. (2023) | COVID-19 | ICU | M/F | 30 | Ultrasound | Anterior Tibialis (right) | Thickness | ↓ | 7 | <0.001 |
| De Moura et al. (2023) | COVID-19 | ICU | M/F | 30 | Ultrasound | Quadriceps (left) | Thickness | ↓ | 7 | 0.012 |
| De Moura et al. (2023) | COVID-19 | ICU | M/F | 30 | Ultrasound | Rectus Femoris (left) | Thickness | ↓ | 7 | 0.002 |
| De Moura et al. (2023) | COVID-19 | ICU | M/F | 30 | Ultrasound | Vastus Intermedius (left) | Thickness | ↓ | 7 | 0.009 |
| De Moura et al. (2023) | COVID-19 | ICU | M/F | 30 | Ultrasound | Medial Gastrocnemius (left) | Thickness | ↓ | 7 | <0.001 |
| De Moura et al. (2023) | COVID-19 | ICU | M/F | 30 | Ultrasound | Lateral Gastrocnemius (left) | Thickness | ↓ | 7 | <0.001 |
| De Moura et al. (2023) | COVID-19 | ICU | M/F | 30 | Ultrasound | Anterior Tibialis (left) | Thickness | ↓ | 7 | <0.001 |
| Dimopoulos et al. (2020) | Cardiac | Surgery | M/F | 9 | Ultrasound | Rectus Femoris | Thickness | ↙ | 5 | 0.729 |
| Dimopoulos et al. (2020) | Cardiac | Surgery | M/F | 9 | Ultrasound | Rectus Femoris & Vastus Intermedius | Thickness | ↙ | 5 | 0.53 |
| Dirks et al. (2015) | Comatose | ICU | M/F | 9 | Muscle Biopsy | Vastus Lateralis | TI & TII Fibre CSA | ↓ | 7 | <0.05 |
| Gerovasili et al. (2009) | Various | ICU | M/F | 13 | Ultrasound | Rectus Femoris | CSA | ↓ | 9 | NR |
| Gerovasili et al. (2009) | Various | ICU | M/F | 13 | Ultrasound | Vastus Intermedius | CSD | ↓ | 9 | NR |
| Gil et al. (2022) | COVID-19 | Medical ward & ICU | M/F | 80 | Ultrasound | Vastus Lateralis | CSA | ↓ | 8 | <0.001 |
| Hadda et al. (2018) | Sepsis | ICU | M/F | 70 | Ultrasound | Thigh | Thickness | ↓ | 7 | 0.003 |
| Hayes et al. (2018)* | Various | Medical & surgical ICU | M/F | 25 | Ultrasound | Rectus Femoris | CSA | ↓ | 20 | <0.001 |
| Hayes et al. (2018) | Various | Medical & surgical ICU | M/F | 25 | Ultrasound | Rectus Femoris | Thickness | ↓ | 20 | <0.001 |
| Hayes et al. (2018) | Various | Medical & surgical ICU | M/F | 25 | Ultrasound | Vastus Lateralis | Thickness | ↓ | 20 | <0.001 |
| Kangalgil et al. (2022)* | Trauma | Surgical ICU | M/F | 35 | Ultrasound | Rectus Femoris | CSA | ↙ | 7 | NR |
| Kangalgil et al. (2024) | Various | ICU | M/F | 44 | Ultrasound | Rectus Femoris | CSA | ↓ | 7 | <0.001 |
| Katari et al. (2018) | Various | ICU | M/F | 100 | Ultrasound | Rectus Femoris | Thickness | ↓ | 7 | <0.001 |
| Kouw et al. (2019)* | Osteoarthritis | Surgery | M/F | 26 | CT | Rectus Femoris | CSA | ↓ | 6 | 0.004 |
| Kouw et al. (2019) | Osteoarthritis | Surgery | M/F | 26 | CT | Quadriceps | Total Thigh Volume | ↓ | 6 | 0.03 |
| Kouw et al. (2019) | Osteoarthritis | Surgery | M/F | 26 | Muscle Biopsy | Vastus Lateralis | TI & TII Fibre CSA | ↗ | 6 | 0.1 |
| Lee et al. (2021)* | Various | Medical & surgical ICU | M/F | 53 | Ultrasound | Rectus Femoris | CSA | ↙ | 6 | NR |
| Lee et al. (2021)* | Various | Medical & surgical ICU | M/F | 24 | Ultrasound | Rectus Femoris | CSA | ↙ | 13 | NR |
| Lee et al. (2021)* | Various | Medical & surgical ICU | M/F | 10 | Ultrasound | Rectus Femoris | CSA | ↙ | 21 | NR |
| Lee et al. (2021) | Various | Medical & surgical ICU | M/F | 86 | Ultrasound | Quadriceps | Thickness | ↓ | 22 | 0.039 |
| Lopez Jiminez et al. (2024)* | Various | Medical ward | M/F | 74 | Ultrasound | Rectus Femoris (right) | CSA | ↙ | 8 | 0.177 |
| Lopez Jiminez et al. (2024)* | Various | Medical ward | M/F | 74 | Ultrasound | Rectus Femoris (left) | CSA | ↙ | 8 | 0.156 |
| Lopez Jiminez et al. (2024) | Various | Medical ward | M/F | 74 | Ultrasound | Rectus Femoris | Thickness | ↓ | 8 | 0.043 |
| Mayer et al. (2020)* | Acute Respiratory Failure | ICU | M/F | 41 | Ultrasound | Rectus Femoris | CSA | ↓ | 7 | 0.025 |
| Mayer et al. (2020) | Acute Respiratory Failure | ICU | M/F | 41 | Ultrasound | Rectus Femoris | Thickness | ↓ | 7 | 0.031 |
| Mayer et al. (2020) | Acute Respiratory Failure | ICU | M/F | 41 | Ultrasound | Tibialis Anterior | CSA | ↓ | 7 | <0.001 |
| Mayer et al. (2020) | Acute Respiratory Failure | ICU | M/F | 41 | Ultrasound | Tibialis Anterior | Thickness | ↓ | 7 | <0.001 |
| McNelly et al. (2020) | Multi-organ Failure | ICU | M/F | 121 | Ultrasound | Rectus Femoris | CSA | ↓ | 10 | NR |
| Nakanishi et al. (2020) | Various | Medical & surgical ICU | M/F | 64 | Ultrasound | Rectus Femoris | CSA | ↓ | 7 | <0.01 |
| Nickels et al. (2020) | Various | Medical & surgical ICU | M/F | 36 | Ultrasound | Rectus Femoris | CSA | ↓ | 10 | NR |
| Nickels et al. (2020) | Various | Medical & surgical ICU | M/F | 36 | Ultrasound | Rectus Femoris | Thickness | ↓ | 10 | NR |
| Nickels et al. (2020) | Various | Medical & surgical ICU | M/F | 36 | Ultrasound | Vastus Intermedius | Thickness | ↓ | 10 | NR |
| Parry et al. (2015) | Various | Medical & surgical ICU | M/F | 22 | Ultrasound | Rectus Femoris | CSA | ↓ | 10 | <0.001 |
| Parry et al. (2015) | Various | Medical & surgical ICU | M/F | 22 | Ultrasound | Rectus Femoris | Thickness | ↓ | 10 | <0.001 |
| Pourhassan et al. (2020) | Various - NOT Malnourished | Medical ward | M/F | 34 | MRI | Mid-Thigh | CSA | ↙ | 14 | NR |
| Pourhassan et al. (2020) | Various - Malnourished | Medical ward | M/F | 7 | MRI | Mid-Thigh | CSA | ↓ | 14 | <0.001 |
| Puthucheary et al. (2013) | Various | Medical & surgical ICU | M/F | 63 | Ultrasound | Rectus Femoris | CSA | ↓ | 10 | <0.001 |
| Puthucheary et al. (2013) | Various | Medical & surgical ICU | M/F | 28 | Muscle Biopsy | Vastus Lateralis | Fibre CSA | ↓ | 7 | NR |
| Rodrigues et al. (2019) | Various | Medical & surgical ICU | M/F | 60 | Ultrasound | Rectus Femoris | CSA | ↓ | 7 | <0.001 |
| Rodrigues et al. (2019) | Various | Medical & surgical ICU | M/F | 60 | Ultrasound | Quadriceps | Thickness | ↓ | 7 | 0.017 |
| Segers et al. (2021) | Various | Medical & surgical ICU | M/F | 47 | Ultrasound | Rectus Femoris & Vastus Lateralis | Thickness | ↓ | 7 | <0.001 |
| Silva et al. (2019) | Traumatic Brain Injury | ICU | M/F | 30 | Ultrasound | Rectus Femoris | Thickness | ↓ | 14 | <0.001 |
| Silva et al. (2019) | Traumatic Brain Injury | ICU | M/F | 30 | Ultrasound | Tibialis Anterior | Thickness | ↓ | 14 | <0.001 |
| Toledo et al. (2021) | Various | ICU | M/F | 74 | Ultrasound | Rectus Femoris (right) | Thickness | ↙ | 7 | NR |
| Toledo et al. (2021) | Various | ICU | M/F | 74 | Ultrasound | Rectus Femoris (left) | Thickness | ↙ | 7 | NR |
| Trung et al. (2019) | Tetanus Infection | ICU | M/F | 80 | Ultrasound | Rectus Femoris | CSA | ↓ | 25 | <0.01 |
| Turton et al. (2016) | Various | ICU | M/F | 9 | Ultrasound | Gastrocnemius | Thickness | ↙ | 10 | 0.72 |
| Turton et al. (2016) | Various | ICU | M/F | 9 | Ultrasound | Vastus Lateralis | Thickness | ↓ | 10 | 0.002 |
| Verceles et al. (2023) | Various | ICU | M/F | 23 | CT | Thigh | CSA | ↙ | 14 | NR |
| Verceles et al. (2023) | Various | ICU | M/F | 23 | CT | Thigh | Volume | ↙ | 14 | NR |
| Verceles et al. (2023) | Various | ICU | M/F | 23 | CT | Lower leg | CSA | ↙ | 14 | NR |
| Verceles et al. (2023) | Various | ICU | M/F | 23 | CT | Lower leg | Volume | ↙ | 14 | NR |
| Xie et al. (2019)* | Various - ICU AW | ICU | M/F | 50 | Ultrasound | Rectus Femoris | CSA | ↙ | 7 | NR |
| Xie et al. (2019)* | Various - Non-ICU AW | ICU | M/F | 45 | Ultrasound | Rectus Femoris | CSA | ↙ | 7 | NR |
| Sex: M = Male, F = Female; NR = Not reported; ICU = Intensive Care Unit; CT = Computed Tomography; MRI = Magnetic Resonance Imaging; CSA = Cross-sectional Area; CSD = Cross-sectional Diameter; TI = Type 1; TII = Type 2; AW = Acquired Weakness; * = included in meta analysis. ↓ = significant reduction; ↙ = non-significant reduction; ↔ = no change; ↗ = non-significant increase; ↑ = significant increase. | | | | | | | | | | |

***Table S8.*** *Summary of changes in muscle function during hospitalisation.*

| **Author(s)** | **Population** | **Exposure** | **Sex** | **Number** | **Measurement Tool** | **Outcome on Muscle Function** | **Duration (days)** | ***p* Value** |
| --- | --- | --- | --- | --- | --- | --- | --- | --- |
| Aarden et al. (2021) | Various | Medical ward | M/F | 343 | SPPB | ↗ | 7 | NR |
| Ahmad et al. (2024)* | Cardiac | Surgery | M/F | 15 | 6MWT | ↙ | 11 | NR |
| Bodilsen et al. (2013)* | Various | Medical ward | M/F | 33 | TUG | ↑ | 8 | 0.003 |
| Borges et al. (2016)* | CABG | Surgery | M/F | 19 | 6MWT | ↓ | 8 | 0.01 |
| Butera et al. (2024)* | Various | Medical ward & Surgery | M/F | 698 | SPPB | ↗ | 19 | NR |
| Butera et al. (2024)* | Various | Medical ward & Surgery | M/F | 444 | Gait Speed | ↗ | 19 | NR |
| De Buyser et al. (2014) | Various | Medical ward | M/F | 639 | Gait Speed | ↑ | 9 | <0.001 |
| De Morton et al. (2007)* | Various | Medical ward | M/F | 126 | TUG | ↗ | 6 | NR |
| Eibel et al. (2022)* | CABG | Surgery | M/F | 6 | 6MWT | ↓ | 7 | 0.006 |
| Fränzel et al. (2024)* | Various | Medical ward | M/F | 29 | SPPB | ↗ | 21 | NR |
| Fränzel et al. (2024)* | Various | Medical ward | M/F | 29 | TUG | ↗ | 21 | NR |
| Fränzel et al. (2024)* | Various | Medical ward | M/F | 29 | Gait Speed | ↗ | 21 | NR |
| Giangregorio et al. (2009)* | Hip Fracture | Surgery | M/F | 7 | TUG | ↗ | 26 | NR |
| Hu et al. (2020)* | Various | Medical ward | M/F | 50 | TUG | ↑ | 7 | NR |
| Jones et al. (2006) | Various | Medical ward | M/F | 80 | TUG | ↗ | 11 | NR |
| Karlsen et al. (2017) | Various | Medical ward | M/F | 151 | CST | ↑ | 9 - 13 | <0.001 |
| Martínez-Velilla et al. (2019) | Various | Medical ward | M/F | 185 | SPPB | ↗ | 8 | NR |
| Martín-Salvador et al. (2015) | Pneumonia (<75 years old) | Medical ward | M/F | 68 | STS | ↓ | 8 | <0.001 |
| Martín-Salvador et al. (2015) | Pneumonia (>75 years old) | Medical ward | M/F | 48 | STS | ↙ | 8 | 0.224 |
| Mayer et al. (2020) | Acute Respiratory Failure | ICU | M/F | 41 | SPPB | ↗ | 6 | NR |
| Nery et al. (2012)* | Lung Resection | Surgery | M/F | 15 | 6MWT | ↓ | 7 | <0.001 |
| Norheim et al. (2017) | Various - Reducing inflammation | Medical ward | M/F | 76 | CST | ↔ | 8 | 0.932 |
| Norheim et al. (2017) | Various - Continual inflammation | Medical ward | M/F | 137 | CST | ↑ | 8 | <0.001 |
| Ortiz-Alonso et al. (2019)* | Various | Medical ward | M/F | 125 | SPPB | ↗ | 7 | NR |
| Ramsey et al. (2022) | Various | Medical ward | M/F | 547 | SPPB | ↑ | 20 | <0.001 |
| Ramsey et al. (2022) | Various | Medical ward | M/F | 548 | CST | ↑ | 20 | <0.001 |
| Ramsey et al. (2022) | Various | Medical ward | M/F | 556 | Gait Speed | ↑ | 20 | <0.001 |
| Raymond et al. (2017) | Various | Medical ward | M/F | 223 | TUG | ↗ | 12 | NR |
| Raymond et al. (2017) | Various | Medical ward | M/F | 232 | Gait Speed | ↗ | 12 | NR |
| Temporiti et al. (2022)* | THA | Surgery | M/F | 40 | TUG | ↙ | 4 | NR |
| Temporiti et al. (2022)* | THA | Surgery | M/F | 40 | Gait Speed | ↙ | 4 | NR |
| Torres-Sánchez et al. (2017) | COPD | Medical ward | M/F | 29 | STS | ↙ | 10 | NR |
| Werner et al. (2024) | Various | Medical ward | M/F | 107 | SPPB | ↑ | 20 | <0.001 |
| Werner et al. (2024) | Various | Medical ward | M/F | 107 | CST | ↑ | 20 | <0.001 |
| Werner et al. (2024) | Various | Medical ward | M/F | 107 | Gait Speed | ↑ | 20 | <0.001 |
| Wnuk et al. (2016) | Abdominal Aortic Aneurysm | Surgery | M/F | 16 | 6MWT | ↙ | 7 | NR |
| Xue et al. (2022)* | Cardiac | Surgery | M/F | 43 | SPPB | ↗ | 8 | NR |
| Xue et al. (2022)* | Cardiac | Surgery | M/F | 43 | 6MWT | ↗ | 8 | NR |
| Sex: M = Male, F = Female; NR = Not reported; CABG = Coronary Artery Bypass Graft; THA = Total Hip Arthroplasty; ICU = Intensive Care Unit; SPPB = Short Physical Performance Battery; TUG = Timed Up-And-Go; 6MWT = 6 Minute Walk Test; CST = Chair Stand Test; STS = Sit-to-stand test. ↓ = significant reduction; ↙ = non-significant reduction; ↔ = no change; ↗ = non-significant increase; ↑ = significant increase. | | | | | | | | |

***Table S9.*** *Summary of changes in muscle quality indices during hospitalisation.*

| **Author(s)** | **Population** | **Exposure** | **Sex** | **Number** | **Measurement Tool** | **Outcome Measure** | **Muscle Assessed** | **Outcome on Muscle Quality** | **Duration (days)** | ***p* Value** |
| --- | --- | --- | --- | --- | --- | --- | --- | --- | --- | --- |
| De Andrade-Juníor et al. (2021) | COVID-19 | ICU | M/F | 32 | Ultrasound | Echogenicity | Rectus Femoris | ↓ | 10 | <0.05 |
| Dusseaux et al. (2019) | Various | ICU | M/F | 25 | CT | Muscle Attenuation | Abdomen | ↙ | 11 | 0.493 |
| Gualtieri et al. (2020) | COVID-19 | ICU | M/F | 30 | CT | Muscle Attenuation | Erector Spinae | ↔ | 20 | 0.51 |
| Gualtieri et al. (2020) | COVID-19 (Obese) | ICU | M/F | 17 | CT | Muscle Attenuation | Erector Spinae | ↔ | 20 | 0.95 |
| Gualtieri et al. (2020) | COVID-19 (Lean) | ICU | M/F | 13 | CT | Muscle Attenuation | Erector Spinae | ↙ | 20 | 0.404 |
| Hayes et al. (2018) | Various | Medical & surgical ICU | M/F | 25 | Ultrasound | Echogenicity | Rectus Femoris | ↙ | 20 | 0.41 |
| Kouw et al. (2019) | Osteoarthritis | Surgery | M/F | 26 | CT | Muscle Attenuation | Erector Spinae | ↓ | 6 | <0.001 |
| Lee et al. (2021) | Various | Medical & surgical ICU | M/F | 86 | Ultrasound | Echogenicity | Rectus Femoris | ↔ | 22 | NR |
| Lee et al. (2021) | Various | Medical & surgical ICU | M/F | 86 | Ultrasound | Pennation Angle | Rectus Femoris | ↙ | 22 | 0.067 |
| Lee et al. (2021) | Various | Medical & surgical ICU | M/F | 86 | Ultrasound | Fascicle Length | Rectus Femoris | ↗ | 22 | 0.49 |
| Mayer et al. (2020) | Acute Respiratory Failure | ICU | M/F | 41 | Ultrasound | Echogenicity | Rectus Femoris | ↙ | 7 | 0.08 |
| Mayer et al. (2020) | Acute Respiratory Failure | ICU | M/F | 41 | Ultrasound | Echogenicity | Tibialis Anterior | ↓ | 7 | 0.002 |
| Parry et al. (2015) | Various | Medical & surgical ICU | M/F | 22 | Ultrasound | Echogenicity | Rectus Femoris | ↙ | 10 | NR |
| Parry et al. (2015) | Various | Medical & surgical ICU | M/F | 22 | Ultrasound | Echogenicity | Vastus Lateralis | ↙ | 10 | NR |
| Silva et al. (2019) | Traumatic Brain Injury | ICU | M/F | 30 | Ultrasound | Echogenicity | Rectus Femoris | ↓ | 14 | <0.05 |
| Silva et al. (2019) | Traumatic Brain Injury | ICU | M/F | 30 | Ultrasound | Echogenicity | Tibialis Anterior | ↓ | 14 | <0.05 |
| Strasser et al. (2023) | Various | Medical ward | M/F | 10 | BIA | Phase Angle | Skeletal Muscle | ↙ | 7 | 0.521 |
| Turton et al. (2016) | Various | ICU | M/F | 9 | Ultrasound | Fascicle Length | Gastrocnemius | ↙ | 10 | NS |
| Turton et al. (2016) | Various | ICU | M/F | 9 | Ultrasound | Fascicle Length | Vastus Lateralis | ↙ | 10 | NS |
| Turton et al. (2016) | Various | ICU | M/F | 9 | Ultrasound | Pennation Angle | Gastrocnemius | ↙ | 10 | NS |
| Turton et al. (2016) | Various | ICU | M/F | 9 | Ultrasound | Pennation Angle | Vastus Lateralis | ↓ | 10 | 0.18 |
| Welch et al. (2022a) | Colorectal surgery & Acute Infection | Medical ward & surgery | M/F | 79 | Ultrasound | Echogenicity | Rectus Femoris | ↙ | 7 | NS |
| Welch et al. (2022a) | Colorectal surgery & Acute Infection | Medical ward & surgery | M | 48 | BIA | Phase Angle | Skeletal Muscle | ↓ | 7 | 0.026 |
| Welch et al. (2022a) | Colorectal surgery & Acute Infection | Medical ward & surgery | F | 31 | BIA | Phase Angle | Skeletal Muscle | ↙ | 7 | NS |
| Sex: M = Male, F = Female; NR = Not reported; NS = Not significant; ICU = Intensive Care Unit; BIA = Bio-electrical Impedance analysis. ↓ = significant reduction; ↙ = non-significant reduction; ↔ = no change; ↗ = non-significant increase; ↑ = significant increase. | | | | | | | | | | |
